# Evolutionary genomic relationships and coupling in MK-STYX and STYX pseudophosphatases

**DOI:** 10.1038/s41598-022-07943-5

**Published:** 2022-03-09

**Authors:** Yi Qi, Di Kuang, Kylan Kelley, William J. Buchser, Shantá D. Hinton

**Affiliations:** 1 Department of Biology, Integrated Science Center, College of William and Mary, 540 Landrum Drive, Williamsburg, VA 23185 USA; 2 Department of Genetics, Washington University, St. Louis, MO 63110 USA

**Keywords:** Biochemistry, Computational biology and bioinformatics, Evolution, Genetics, Molecular biology

## Abstract

The dual specificity phosphatase (DUSP) family has catalytically inactive members, called pseudophosphatases. They have mutations in their catalytic motifs that render them enzymatically inactive. This study analyzes the significance of two pseudophosphatases, MK-STYX [MAPK (mitogen-activated protein kinase phosphoserine/threonine/tyrosine-binding protein]) and STYX (serine/threonine/tyrosine-interacting protein), throughout their evolution and provides measurements and comparison of their evolutionary conservation. Phylogenetic trees were constructed to show any deviation from various species evolutionary paths. Data was collected on a large set of proteins that have either one of the two domains of MK-STYX, the DUSP domain or the cdc-25 homology (CH2) /rhodanese-like domain. The distance between species pairs for MK-STYX or STYX and Ka/Ks ratio were calculated. In addition, both pseudophosphatases were ranked among a large set of related proteins, including the active homologs of MK-STYX, MKP (MAPK phosphatase)-1 and MKP-3. MK-STYX had one of the highest species-species protein distances and was under weaker purifying selection pressure than most proteins with its domains. In contrast, the protein distances of STYX were lower than 82% of the DUSP-containing proteins and was under one of the strongest purifying selection pressures. However, there was similar selection pressure on the N-terminal sequences of MK-STYX, STYX, MKP-1, and MKP-3. We next perform statistical coupling analysis, a process that reveals interconnected regions within the proteins. We find that while MKP-1,-3, and STYX all have 2 functional units (sectors), MK-STYX only has one, and that MK-STYX is similar to MKP-3 in the evolutionary coupling of the active site and KIM domain. Within those two domains, the mean coupling is also most similar for MK-STYX and MKP-3. This study reveals striking distinctions between the evolutionary patterns of MK-STYX and STYX, suggesting a very specific role for each pseudophosphatase, further highlighting the relevance of these atypical members of DUSP as signaling regulators. Therefore, our study provides computational evidence and evolutionary reasons to further explore the properties of pseudophosphatases, in particular MK-STYX and STYX.

## Introduction

The phosphorylation cascade is a critical component of signal transduction; the coordination of kinases and phosphatases is essential for regulation. The pseudoenzymes pseudokinases and pseudophosphatases have introduced another level of complexity and regulation, which is less understood. Pseudoenzymes are catalytic impaired due to mutations that result in an absence of critical residues^1–3^; though their three dimensional fold is maintained^1^. Pseudoenzymes are within more than twenty enzyme families^3^, and approximately ten percent of the proteins are considered pseudoenzymes^1–4^.

Fourteen percent of members of the phosphatase family are pseudophosphatases^3,4^. Pseudophosphatases have emerged as critical regulators of signaling pathways^3,4^. They exert their function by serving as competitors, signaling integrators, modulators, and anchors in cellular processes^3–5^. In addition, pseudophosphatase roles have been implicated in various human diseases^3,6^. Recently, there has been an explosion of data implicating the pseudophosphatase MK-STYX [MAPK (mitogen-activated protein kinase) phosphoserine/threonine/tyrosine-binding protein] in diseases such as hepatocellular carcinoma^7^ and glioblastoma^6,8^. Pseudophosphatases roles as signaling regulators and linkage to diseases indicate their immediate importance to understanding their molecular mechanism(s).

Most enzymes have catalytically inactive homologs, which are highly conserved^1^. We apply a bioinformatics approach to understand the evolutionary genomic relationship of two pseudophosphatases, MK-STYX and STYX. The MK-STYX protein is encoded by the gene *STYXL1* (serine/threonine/tyrosine interacting like 1) and also is referred to as DUSP24 (dual specificity phosphatase 24). There have been a few studies on evolutionary history of STYXL1^3,9–12^. These studies provided significant contributions to our knowledge about MK-STYX such as revealing the point mutations and its appearance in evolutionary history. However, the structure–function relationship of molecules is vital to understanding the mechanism of any protein’s interactions. Protein interactions and function can be inferred through comparative and evolutionary genetics, which are pursued in this manuscript. Large scale bioinformatics studies such as usage of gene clusters to infer functional coupling are imperative in understanding the molecular mechanism of pseudophosphatase^3,13^. New types of analyses and better models for calculating co-evolution and interacting networks have been developed^14^, which has expanded our knowledge of the function of proteins.

While MK-STYX is atypical, its protein domains are quite common. MK-STYX is the pseudophosphatase member of the MAPK phosphatase (MKP) subfamily, which negatively regulates MAPKs^15–17^. There are eleven mammalian members (ten catalytically active MKPs and one atypical, MK-STYX) of the MKP subfamily^4,15,16,18–20^. MK-STYX has a mutation in the active signature motif HCX_5_R; in which the histidine is replaced by a phenylalanine and the essential cysteine replaced by serine^9,19,21^. Nonetheless all MKP members possess a C-terminal catalytic phosphatase domain and an N-terminal non-catalytic domain composed of two CDC25 (cell division cycle 25)/rhodanese homology (CH2/rhodanese) domains^16,20,22,23^. The C-terminal DUSP domain has conserved aspartic acid, arginine, and cysteine residues within the catalytic active site, while the N-terminal non-catalytic domain has intervening clusters of basic amino acids^15,24^. However, MK-STYX also has a mutation within the N-terminal domains. MK-STYX is mutated in the kinase interaction motif (KIM) domain, which requires consecutive critical arginine residues required for MAPK/ERK docking^44^. MK-STYX lacks these arginines^16,17,25^.

Here, we ascertain how MK-STYX compares to other proteins that contain the same domains. We compare these intra-protein interactions between this pseudophosphatase and some of its closest relatives, their active MKP homologs. In addition, we analyze the evolutionary difference between MK-STYX and STYX, the prototypical pseudophosphatase. STYX is the first catalytically inactive DUSP characterized^8^, with a glycine residue in place of the essential active-site cysteine^18^. Phylogenetic analysis demonstrated the expressing of MK-STYX in 347 species, ranging from the order of Primates, with a few exceptions, to the class Actinopteri (ray-finned fish). Prototypical STYX was expressed in 419 species ranging from primates to Actinopterygii (and birds). MK-STYX and STYX also showed similar selection pressure on the N- and C-terminal sequences. In addition, MK-STYX had one of the highest species-species protein distances, and was under weaker purifying selection pressure than most proteins with its domains. However, the protein distances of STYX were lower than 82% of the DUSP-containing proteins, and was under one of the strongest purifying selection pressures. In addition, we implemented an additional type of analysis, statistical coupling analysis (SCA), which determines which amino acids are evolutionarily linked^26^. Furthermore, SCA has been used to identify functional regions within the protein and how these regions might interact with each other. SCA analysis demonstrates that MK-STYX diverged from its active homolog in a manner where its residues in the active site are mostly one sector, suggesting a rational for MK-STYX’s numerous functions. This study reveals striking distinctions between the evolutionary patterns of MK-STYX and STYX, suggesting a very specific role for each pseudophosphatase. This study further highlights the relevance of these atypical members of DUSP as signaling regulators providing evolutionary rational to further explore the properties of pseudophosphatases.

## Results

### Phylogenetic variations of MK-STYX and STYX

**Figure 1:**
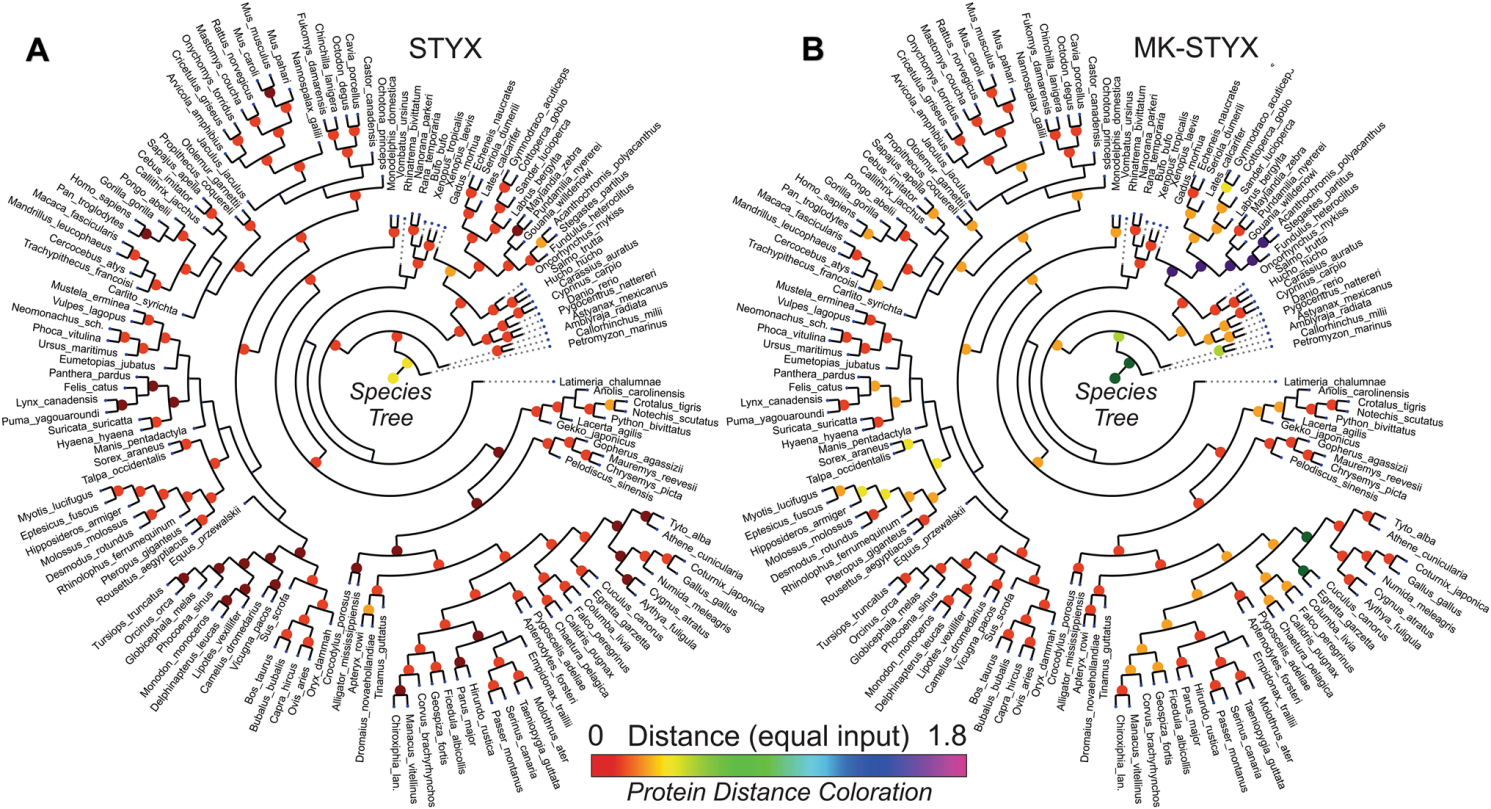
Distance relationships on a phylogenetic tree for STYX and MK-STYX. Phylogenetic tree for 145 of the organisms in our dataset. The tree’s structure is based on standard phylogeny. The tree was constructed using species-species differences (both trees are identical), while the markers plotted over the tree are indicating the protein distances. Nodes are colored on the distance of species-species comparison of the protein sequence (using the equal input model) for STYX (**A**) and MK-STYX (**B**). Cooler colors indicate more divergent sequences and warmer colors indicate more closely related sequences. MK-STYX protein sequence tends to vary greatly when comparing species or clades around the tree. Phylogenetic trees were generated by python (https://www.python.org.) and phyloT^52^ (https://phylot.biobyte.de/index.cgi) and analyzed and displayed with iTOL^51^ (version 1.0) https://itol.embl.de/ and environment for tree exploration (ETE3)^56^ (http://etetoolkit.org/).

Understanding the genomic variation between organisms is a powerful way to understand the relationship between genes and biological characteristics^27^. The phylogenetic relationships between organisms may be determined and analyzed through a phylogenetic clade. First, the phylogenetic variation between species that express MK-STYX and STYX were determined (Supplemental Fig. S1). The exemplary phylogenetic trees of STYX and MK-STYX contain 145 species, including 71 species of mammals, 14 of which are primates, 30 birds, 26 fish, 12 reptiles, and 6 amphibians. Next, we used the standard tree-of-life (refined to 145 species) to show the underlying relationships between the organisms, but then overlaid the evolutionary differences of just MK-STYX or STYX onto the tree. MK-STYX is more divergent than STYX as seen by the protein distance comparisons with larger protein distance values in MK-STYX than in STYX (Fig. 1A,B). The distances on the phylogenetic tree corresponding to STYX show stronger conservation of the gene comparing different species, with most nodes showing very warm colors, indicating small distances between the species of that node. MK-STYX, however, has quite large protein distances. For example, *actinopterygii* have small distance values between them in STYX but are more distant in MK-STYX as shown by the cool colors of their nodes. This observation is also noted with *carnivora*, with the corresponding nodes of STYX having some of the warmest colors, the exact opposite in MK-STYX. Lastly, it is interesting to note that some of the strongest conservation across species occurs in marine mammals, which can be seen in both STYX and MK-STYX, the clearest example where strong conservation is seen for both proteins (Fig. 1A,B).

### MK-STYX has lower protein-sequence conservation than STYX

**Figure 2:**
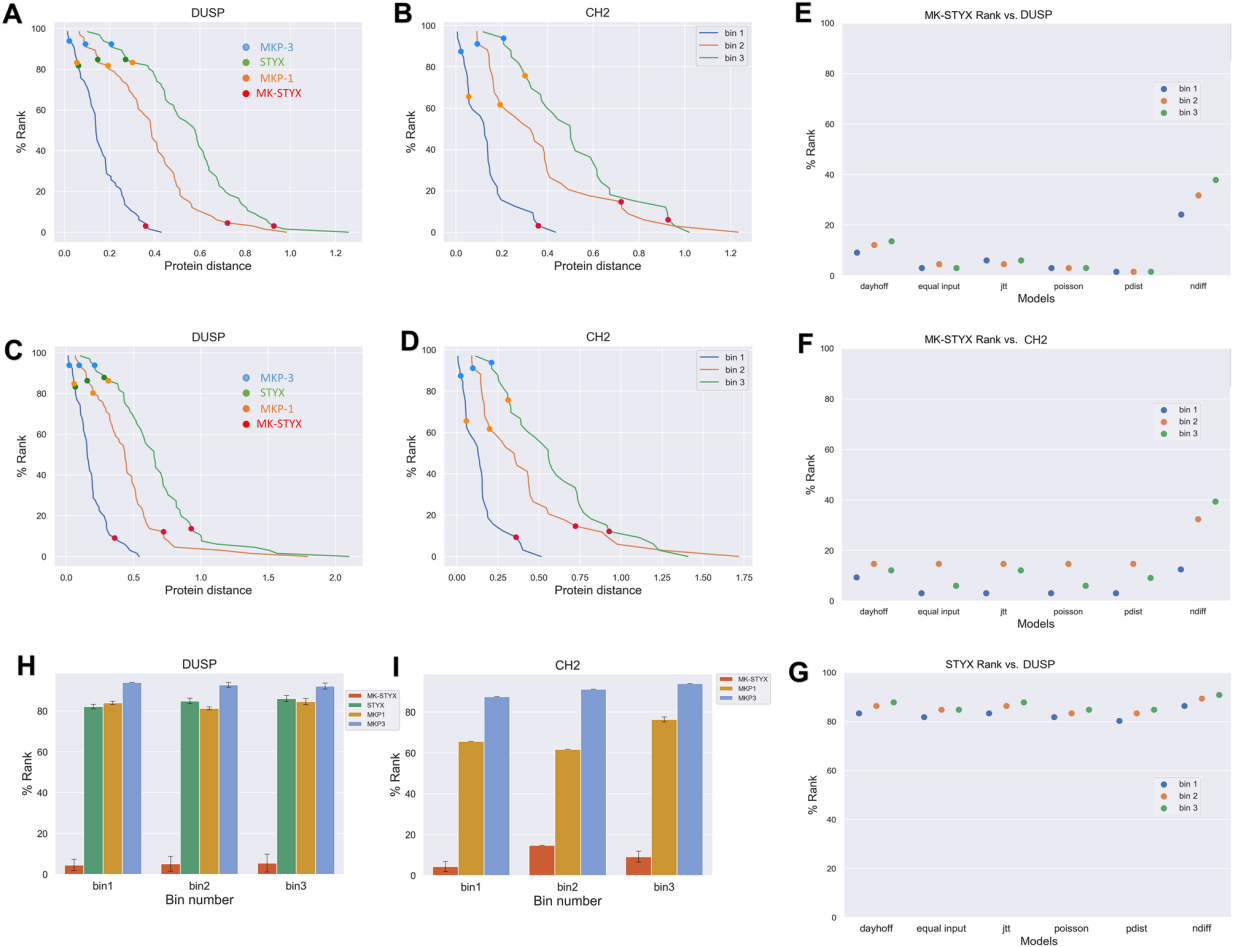
Ranks of the mean distances of MK-STYX, STYX, MKP-1, and MKP-3 by different models. (**A**) Cumulative probability histogram representing the mean distance of proteins with the DUSP domain as calculated by the equal input model in the first three bins. The rank of MK-STYX is indicated by a red circle, the rank of MKP-3 is indicated by a blue circle, the rank of STYX is indicated by a green circle, and the rank of MKP-1 is indicated by an orange circle. (**B**) Cumulative probability histogram (as in **A**) of proteins with the CH2 domain. The rank of MK-STYX is indicated by a red circle, the rank of MKP-3 is indicated by a blue circle, and the rank of MKP-1 is indicated by an orange circle. (**C**) Cumulative probability histogram representing the mean distance of proteins with the DUSP domain as calculated by the Dayhoff model in the first three bins. The rank of MK-STYX is indicated by a red circle, the rank of MKP-3 is indicated by a blue circle, the rank of STYX is indicated by a green circle, and the rank of MKP-1 is indicated by an orange circle. (**D**) Cumulative probability histogram (as in **C**) of proteins with the CH2 domain. The rank of MK-STYX is indicated by a red circle, the rank of MKP-3 is indicated by a blue circle, and the rank of MKP-1 is indicated by an orange circle. (**E**) Dot plot showing the percentile rank of MK-STYX in the DUSP group by six different models. Ranks in the first three bins are shown. (**F**) Dot plot (as in **E**) of MK-STYX in the CH2 group. (**G**) Dot plot showing the percentile rank of STYX in the DUSP group by six different models. Ranks in the first three bins are shown. (**H**) Bar chart showing the mean percentile rank of the distances of MK-STYX, STYX, MKP-1, and MKP-3 calculated by different models in the first three bins. Error bars are the standard deviations of bins. (**I**) Bar chart (as in **F**) of MK-STYX, MKP-1, and MKP-3. Python (Core Team 2015; https://www.python.org.) packages Matplotlib (version2.2.2; https://doi.org/10.5281/zenodo.1202077) and Seaborn^57^ were used to plot and analyze data. The layout was designed in Microsoft PowerPoint.

The conservation pattern of MK-STYX protein sequences was analyzed to determine whether it was more like other DUSP-domain containing proteins (including STYX), or other CH2-domain containing proteins. Our dataset included 36 CH2-domain containing proteins and 68 DUSP-domain containing proteins, including most of the proteins analyzed in the human phosphatome project^28^. For each protein, the evolutionary distance between every pair of species was calculated and put into a distance matrix (for the same protein, i.e. only comparing the evolutionary ‘distance’ between homologs, explained extensively in^29^). Three bin borders were determined from the frequency distribution of the mean protein distances of all species-species pairs (Supplemental Fig. S2). These bins ensured grouping of species pairs that have similar distances, avoiding comparison between closely related species pairs to distant ones. In each bin, the mean pairwise distance of every protein was calculated with six models and ranked by its percentile in that bin. A protein with a lower percentile has higher distances among species. Thus, this protein is changing rapidly, while one with a higher percentile has lower distances and higher sequence conservation. Rankings of MK-STYX species-species distances compared with other DUSP-domain containing proteins (Fig. 2A,C) or compared with CH2-domain containing proteins (Fig. 2B,D) were calculated by two different models. Strikingly, out of all proteins in either grouping, using either model, MK-STYX ranked in the top 10th percentile. Analysis by four additional evolutionary distance models (JTT, p-distance, Dayhoff, Poisson) demonstrate the consistency of these findings (Fig. 2E,F). Thus, the protein divergence of MK-STYX from one species to another species is greater than 90% of the inter-species distance of both other DUSP-containing proteins and the CH2-containing proteins, suggesting the functional differentiation of MK-STYX.

In contrast, the prototypical pseudophosphatase STYX demonstrates high protein sequence conservation, and therefore low species-species distance (Fig. 2A–D). The equal input model shows that the distances of STYX range from the 79th to 87th percentile. All six models demonstrate that the protein sequence of STYX changes much slower than about 82% of other DUSPs (Fig. 2G), suggesting that it is either protected from or independent of traditional DUSP domain evolutionary changes.

Because MKP-1 (gene symbol: *DUSP1*) and MKP-3 (gene symbol: *DUSP6*) are active homologs to MK-STYX, and MKPs and STYX each regulate the MAPK/ERK pathways, the protein conservation among MK-STYX, STYX, MKP-1, and MKP-3 was analyzed (Fig. 2H,I). As expected, MKP-1 and MKP-3 both rank at high percentiles in both the DUSP group and the CH2 group, demonstrating that they are among the slowest to diverge among these domain-containing proteins. Intriguingly, MKP-1 ranked lower than STYX at ~ 75th percentile, suggesting that it is slightly more divergent than STYX—further demonstrating the high conservation of STYX. Furthermore, the fact that MK-STYX is evolving much faster than its active homologs and another DUSP pseudophosphatase may provide insights on its functional divergence from them.

### MK-STYX is under weaker purifying selection than STYX

The mutation rates of these genes were investigated to determine the type of selection at work. Ka is defined as nonsynonymous mutations per nonsynonymous site; Ks is defined as synonymous mutations per synonymous site. Thus, if Ka is greater than Ks (Ka/Ks > 1), then that site or gene is under positive selection because mutations of that region are in favor of amino acid changes. However, if Ka is smaller than Ks (Ka/Ks < 1), then that site or gene is under purifying selection because the resulting protein is largely preserved.

**Figure 3:**
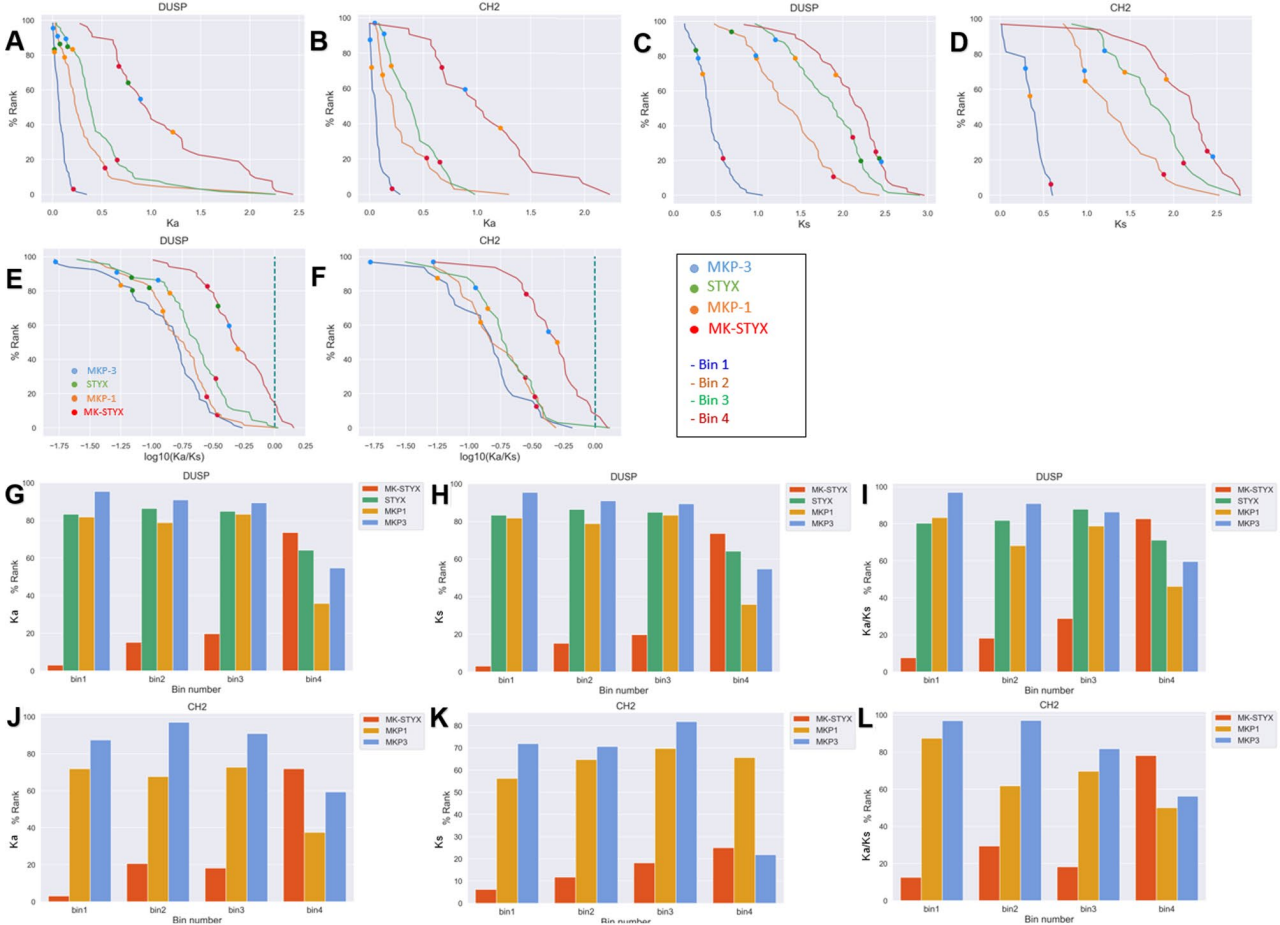
Ranks of median Ka, Ks, and log10(Ka/Ks) of MK-STYX, STYX, MKP-1, and MKP-3 by different models. (**A**) Cumulative probability histogram representing the median Ka of genes with the DUSP domain in the first four bins. The rank of MK-STYX is indicated by a red circle, the rank of MKP-3 is indicated by a blue circle, the rank of STYX is indicated by a green circle, and the rank of MKP-1 is indicated by an orange circle. (**B**) Cumulative probability histogram (as in **A**) of the CH2 domain. (**C**) Cumulative probability histogram representing the median Ks of genes with the DUSP domain in the first four bins. The rank of MK-STYX is indicated by a red circle, the rank of MKP-3 is indicated by a blue circle, the rank of STYX is indicated by a green circle, and the rank of MKP-1 is indicated by an orange circle. (**D**) Cumulative probability histogram (as in **C**) of the CH2 domain. (**E**) Cumulative probability histogram representing the median log10(Ka/Ks) of genes with the DUSP domain in the first three bins. The rank of MK-STYX is indicated by a red circle, the rank of MKP-3 is indicated by a blue circle, the rank of STYX is indicated by a green circle, and the rank of MKP-1 is indicated by an orange circle. The green dash line represents neutral selection pressure, where log_10_(Ka/Ks) = 0 (or Ka = Ks). (**F**) Cumulative probability histogram (as in **E**) of the CH2 domain. (**G**) Bar chart showing the percentile ranks of the median Ka values of MK-STYX, STYX, MKP-1, and MKP-3 in the three bins of the DUSP group. (H) Bar chart (as in **G**) showing the ranks of the median Ks values. (**I**) Bar chart (as in **G**) showing the ranks of the median log_10_(Ka/Ks) values. (**J**) Bar chart showing the percentile ranks of the median Ka values of MK-STYX, MKP-1, and MKP-3 in the three bins of the CH2 group. (**K**) Bar chart (as in **J**) showing the ranks of the median Ks values. (**L**) Bar chart (as in **J**) showing the ranks of the median log_10_ (Ka/Ks) values. Python (Core Team 2015; https://www.python.org.) packages Matplotlib (version2.2.2; https://doi.org/10.5281/zenodo.1202077) and Seaborn^57^ were used to plot and analyze data. The layout was designed in Microsoft PowerPoint.

In each species-species bin (identical to Fig. 2), all pairwise Ka, Ks, and log_10_(Ka/Ks) values were aggregated to produce the corresponding median values and percentile rankings of every gene (Fig. 3A–D). We calculated Ka and Ks (nonsynonymous and synonymous mutation rates, Fig. 3A–D) for all species-species pairs for every protein in the dataset (species-species bins were identical to Fig. 2). To group species-species pairs that are either closely related, very divergent, or those in between bins were created. For example, related species-species pairs are grouped as bin 1, very divergent species-species pairs grouped as bin 4. A more detailed description is available in^30^. The median Ka/Ks ratios of the DUSP and CH2 domains suggest that these two domains are both under purifying selection (Fig. 3E,F). The Ka/Ks ratios were used to compare MK-STYX and STYX with structurally related proteins. Consistent with the evolution of its protein sequences, MK-STYX has coding sequences that are changing faster than most DUSP- and CH2-containing proteins. Depending on the bins, the Ka/Ks ratios for MK-STYX hovered around the 10–20th percentiles in both protein groups. These rankings are attributed to the high Ka values of MK-STYX (Fig. 3A,B) coupled with Ks values in the middle percentiles (Fig. 3C,D) suggesting that the coding sequences of MK-STYX are exposed to higher rates of nonsynonymous substitutions than around 80% of other proteins with the two domains. However, the Ks values of MK-STYX range between 5 and 40% in both groups (Fig. 3C,D) and these moderate synonymous substitution rates of MK-STYX indicate that the great divergence of MK-STYX is not because it has high mutation rate at every substitution site, but because it is likely under weaker purifying evolutionary pressure. Intriguingly, the mutation rates are not consistent across all species-species comparisons, likely indicating different selective pressures in different clades.

The selection pressure on STYX also displays stark contrast to that of MK-STYX. Depending on the bins, the Ka/Ks values of STYX rank from 80 to 95% (Fig. 3E,F), demonstrating that the purifying selection pressure on STYX is strong compared to other DUSPs. Furthermore, while Ka is consistent across species (Fig. 3A,B), Ks varies depending on the species comparisons (Fig. 3C,D). Taken together, STYX is under strong evolutionary pressure to conserve both coding sequences and its translational product.

Figures 3G,J are a comparison of MK-STYX, STYX, MKP-1, and MKP-3 in terms of their Ka values. Similar to the relationships among their protein conservations, the Ka values of MKP-3 ranked at the highest percentile (~ 95%) among the four DUSPs, and the Ka rankings of STYX (~ 82%) were comparable to those of MKP-1 (~ 79%). However, all three ranked much higher than MK-STYX (~ 10%). The rankings are similarly distributed for the Ks of these proteins (Fig. 3H,K). Furthermore, STYX, MKP-3, and MKP-1 are under similarly strong purifying selection (Fig. 3I,L), consistent with the analysis of their protein sequences (Fig. 2). Overall, MK-STYX is under weaker purifying selection than STYX and the active homologs, further validating its active evolutionary changes at the coding level (Fig. 3I,L).

### The selection pressure on MK-STYX’s domains and motifs are nearly neutral

**Figure 4:**
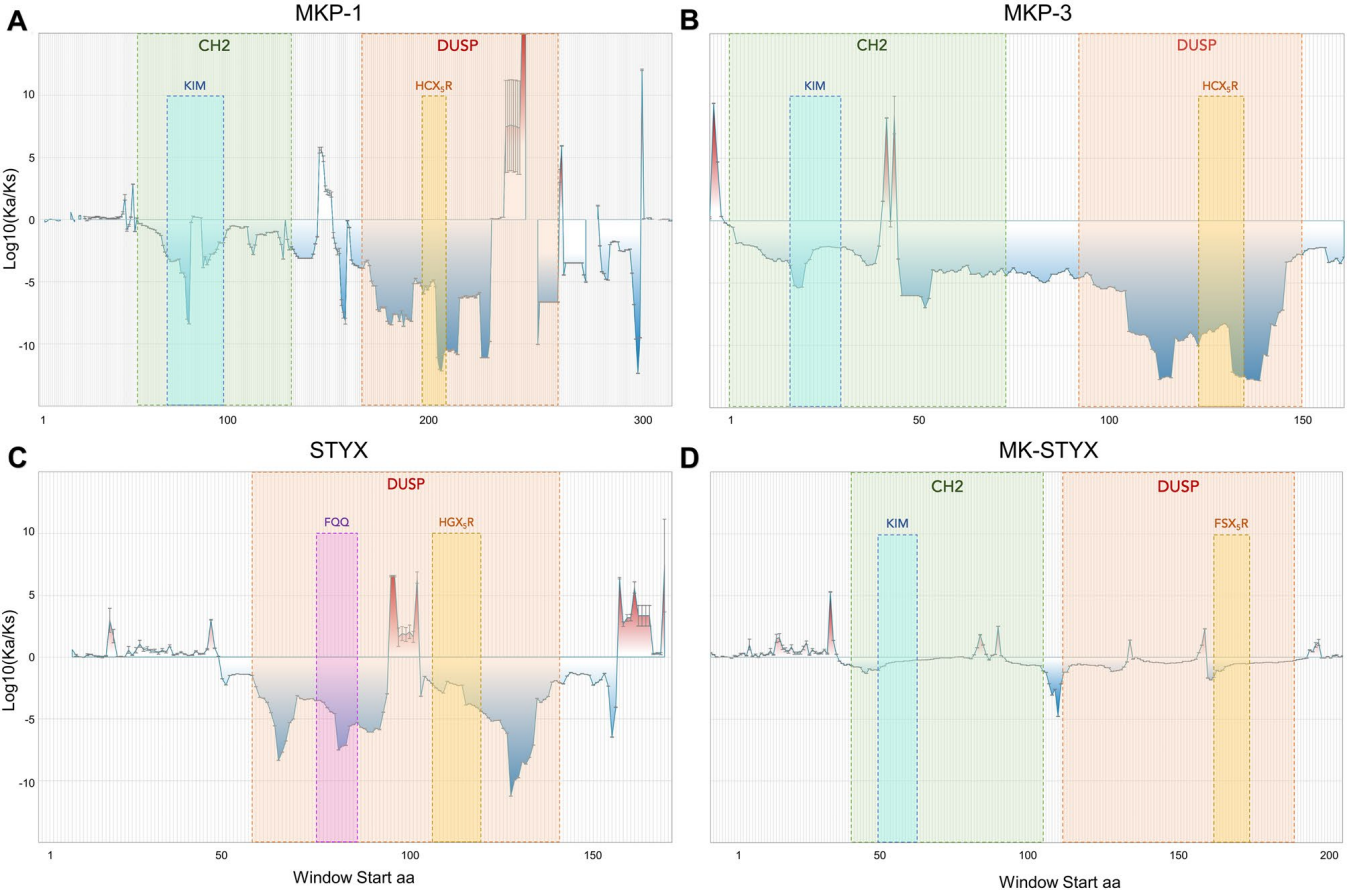
Iceberg plots for Ka, Ks windows of MKP-1, MKP-3, STYX, and MK-STYX. (**A**) Iceberg plot showing the selection pressure acting upon the ‘windows’, motifs, and domain(s) of MKP-3. The window positions of MKP-1’s domains and motifs are listed as the following: CH2 50–127, DUSP 162–260, KIM 65–93, HCX5R 192–204. (**B**) Iceberg plot (as in **A**) for MKP-1. Window positions: CH2 6–78, DUSP 97–155, KIM 22–35, HCX5R 128–140. (**C**) Iceberg plot (as in **A**) for STYX. Window positions: DUSP 58–140, FQQ 75–85, HGX5R 107–119. (**D**) Iceberg plot (as in **A**) for MK-STYX. Window positions: CH2 48–112, DUSP 119–196, KIM 57–70, FSX5R 169–181.

We next sought to examine the evolutionary pressure on regions of the coding sequences in these phosphatases. We generated 99-mer nucleotide regions on the full alignments of MK-STYX, STYX, MKP-1, and MKP-3; every 99-mer window was 9 base pair apart from the proceeding one. Then the Ka and Ks values of all the windows of the four genes of interests were calculated. The regional values of the Ka/Ks ratios (per 99-bp ‘windows’) are plotted in an ‘iceberg’ graph for MKP-1, MKP-3, STYX, and MK-STYX (Fig. 4A–D respectively). The active homologs display similar patterns in their CH2 and DUSP domains. For MKP-1 and MKP-3, the DUSP domains are under stronger purifying selection pressure than the CH2 domains. There is a negative spike in regions that contain the KIM of both proteins, and the regions with the active sites is one of the most conserved regions (Fig. 4A,B). In comparison, selection pressure on the CH2 and DUSP domain of MK-STYX are nearly neutral, without any distinguishable pattern (Fig. 4D). While the DUSP domain of STYX is under similar selection pressure as MKP-1, its active site is much more neutral than the active phosphatase (Fig. 4C). However, the active site of STYX is still under stronger purifying selection pressure than that of MK-STYX. Taken together, the weaker selection pressure on the active sites and the KIM of pseudophosphatases may indicate that other regions of the protein (the intra domain region for example, are under more selective pressure and are more important for the protein’s function. Intriguingly, the N-terminal sequences of all four proteins display similar patterns with most under positive selection. This striking result could potentially invite more future experiments to explore the biological significance of the actively evolving N-terminal sequences of phosphatases. Furthermore, the FQQ motif of STYX include a negative spike, showing that the function of this motif to interact with the F-box protein FBXW7 is under strong purifying selection pressure to be conserved (Fig. 4C)^31^.

### Evolutionary coupling analysis

Up to now, we considered only a single position as being conserved, but proteins don’t evolve each position independently; instead, the interaction between residues is what shapes a protein’s function. Therefore, we utilized statistical coupling analysis (SCA)^26^ to map the interactions within each of these proteins. The first step of SCA is to obtain a covariance matrix of positional covariation around each position in each protein (Supplemental Fig. S3), showing which residues of the protein have co-evolved. Next, spectral decomposition is used to determine how the covariation relates to regions and to define independent components (ICs). We then identify significant eigenmodes from the covariance matrix as the top ICs (Supplemental Fig. S4). MKP-1, MKP-3, and STYX have 7 ICs and MK-STYX has 9 ICs. Finally, we confirm the orthogonality of the top ICs (Supplemental Fig. S5).

**Figure 5:**
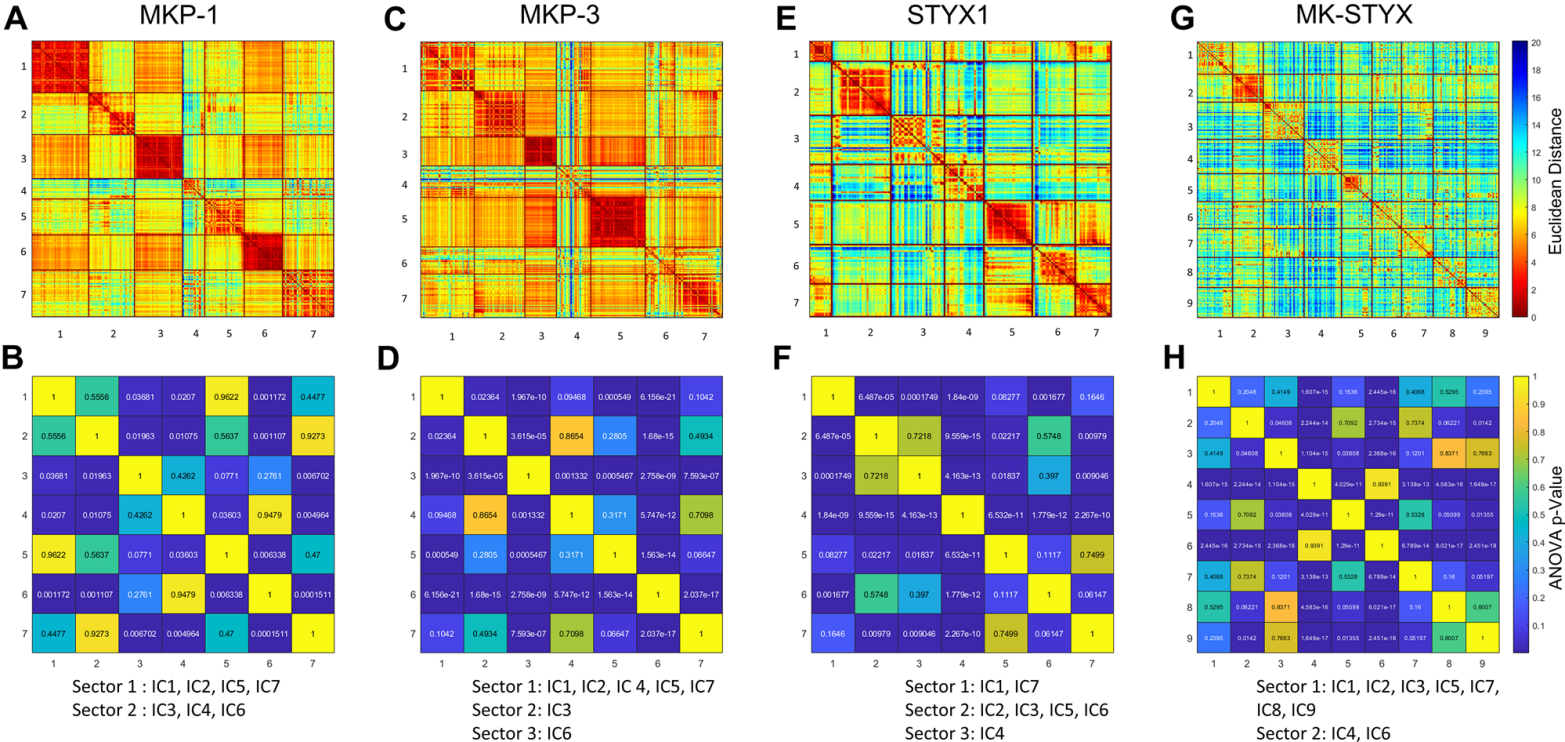
IC heatmap and sector membership analysis for the MKPs, STYX, and MK-STYX. (**A**,**C**,**E**,**G**) The heatmap shows the Euclidean distance between the 7 (MKP-1, MKP-3, or STYX1) or 9 (MK-STYX) selections (top 5% of positions) from 7 ICs or 9 ICs. A hotter color such as red, yellow and orange indicates a strong correlation (short Euclidian distance), whereas a colder color such as green or blue indicates a weak correlation (far Euclidian distance). (**B**,**D**,**F**,**H**) With a large number of residues and indistinguishable correlation coloring among the 7 or 9 selections, an ANOVA test is run between every pair of selections to determine the difference between selections and therefore, to determine sectors. A small enough p-value results in most ICs merging into a giant sector, since most proteins serve one function as an entirety. Thus, a p-value cutoff of 0.1 instead of 0.05 is chosen to prevent excessive merging of ICs. If two selections have a p-value smaller than the cutoff, they are statistically different and should be assigned to different sectors. If two selections have a p-value equal to or bigger than the cutoff, they are not statistically different and should be assigned to the same sector. Two sectors were defined. Heatmaps were generated by MATLAB (https://www.mathworks.com/downloads/web_downloads/download_release?release=R2021a) and the layout was designed in Microsoft PowerPoint.

**Figure 6:**
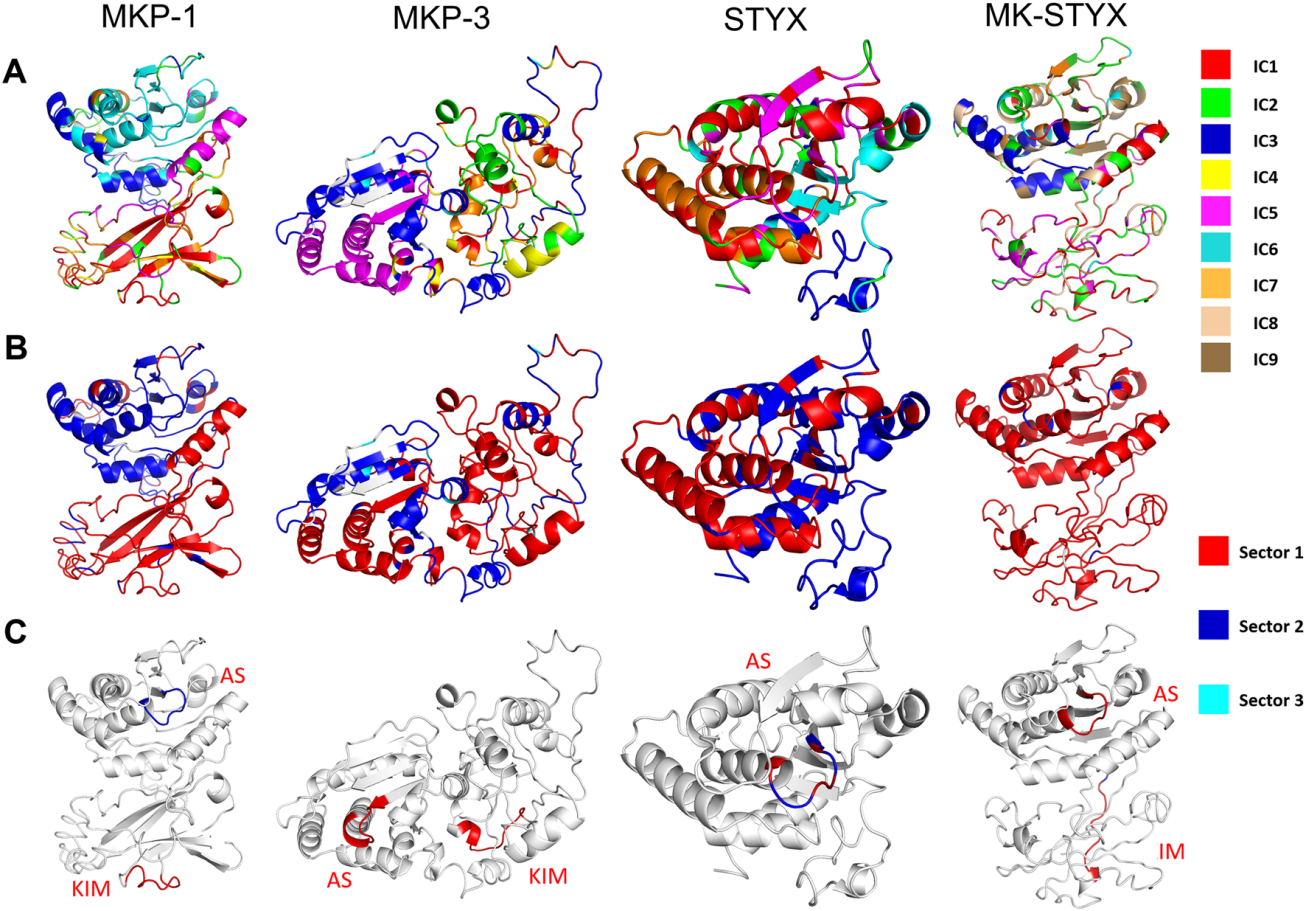
Protein Structure with ICs and sectors from statistical coupling analysis. In each column there are 3 rows, (**A**) shows the protein structure with coloring based on ICs, (**B**) shows the same sequence with sector coloring, and (**C**) has the same structure highlighting only the two motifs, AS (active site), and KIM (kinase interacting motif), each colored with the corresponding sector from above. The columns are for one of each of the proteins studied here (MKP-1, MKP-3, STYX, or MK-STYX. The structural integrity is conserved within the active motif of the active PTPs and pseudophosphatases. However, the KIM domain of MK-STYX has an altered shape. Predictive model of the macromolecular structure of proteins were generated by Iterative Threading ASSEmbly Refinement (I-TASSER) (https://zhanggroup.org/I-TASSER/)^54^. Structures were colored by PyMOL Molecular Graphics System 4.6.0 (https://pymol.org/installers/PyMOL-2.3.3_0-Windows-x86_64.exe) and layout designed in Microsoft PowerPoint.

The ICs are used to define the functional units of the analysis, termed ‘sectors’. To better visualize the relationships of the residues in each IC, heatmaps of the Euclidean distances among selections from ICs were generated (Fig. 5A,C,E,G). Here, the relationships between the ICs are evaluated so that one or more ICs can be combined into related sectors. We formalized this additional level of clustering by generating ANOVA p-values and using them as cut-offs for sector definition (Fig. 5B,D,F,H). ICs with p $\le$ 0.1 were grouped into unique sectors. In Fig. 6, we show the structure of the 4 proteins colored by the ICs (Fig. 6A) and by the sectors (Fig. 6B). For example, we defined two sectors for MKP-1, Sector 1, which includes the kinase interacting motif (KIM): [IC1, IC2, IC5, IC7] and Sector 2, which contains the active site: [IC3, IC4, IC5]. For MKP-3, S1 (AS/KIM): [IC1, IC2, IC4, IC5, IC7], S2: [IC3], and S3: [IC6]. The prototypical pseudophosphatase STYX S1: [IC1, IC7], S2: [IC2, IC3, IC5, IC6], S3: [IC4], and both sector 1 and 2 participate within the active site. Pseudophosphatase MK-STYX has two sectors S1: [IC1, IC2, IC3, IC5, IC7, IC8, IC9] and S2: [IC4, IC6]. Intriguingly, the active site and KIM are within the same sector, Sector 1 (Fig. 6C).

**Figure 7:**
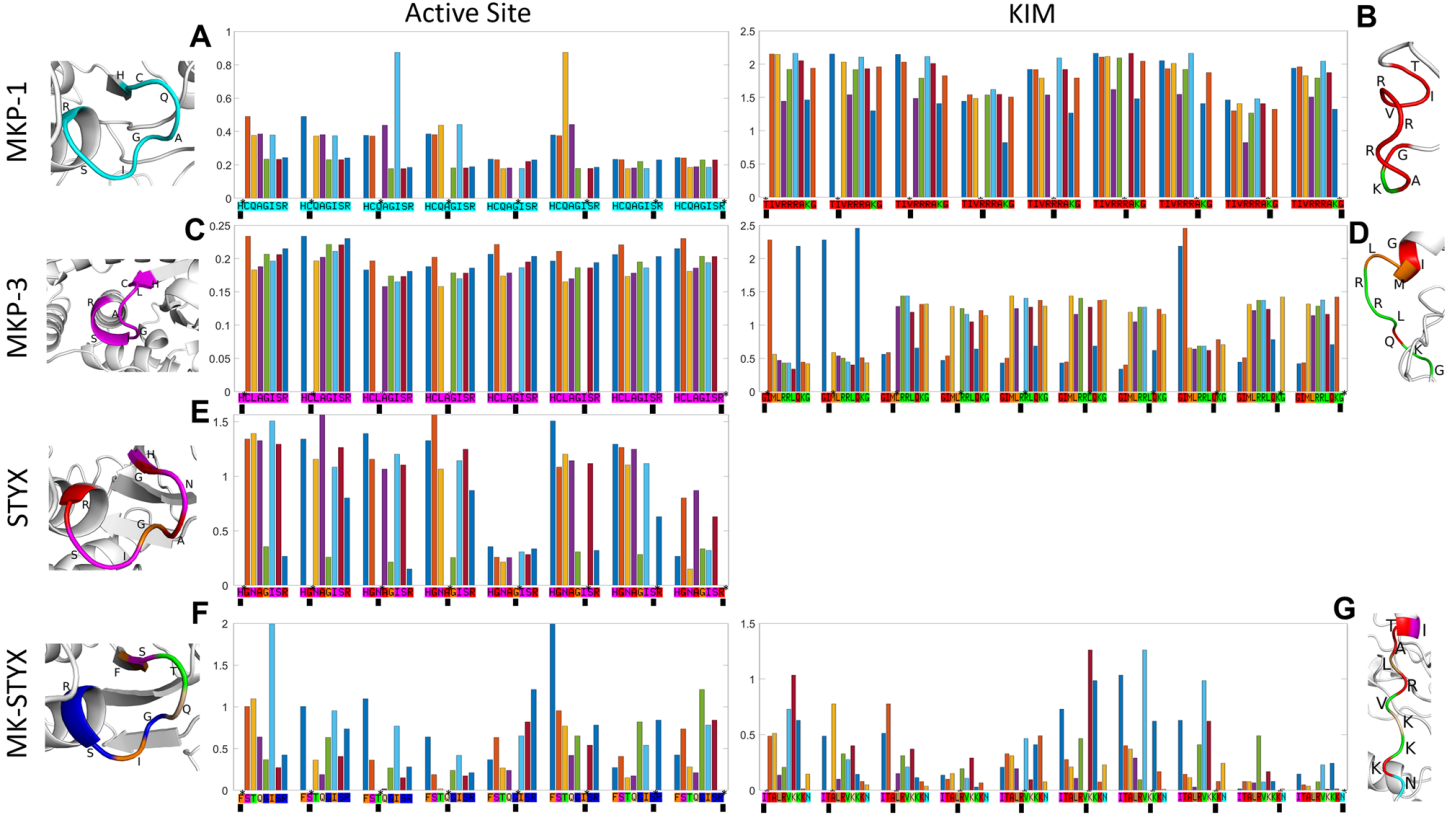
Positional correlations within the active sites and KIM. Each sub-figure shows the positional covariance between every selected position (shown with an asterisk next to the position and a block below it) and all other positions within the active site or kinase interaction domain (KIM) of a given protein. Given any residue, a higher value of covariance of another residue’s indicates a strong correlation, implying coevolution. The covariance value of the selected residue with itself removed (absence of a bar) to avoid visual clutter. The amino acids are labeled under the bars and colored by their IC membership. Next to each sub-figure is the 3-D structure within the active site or the kinase interaction domain. (**A**) Positional correlation of positions within the active site of MKP-1. All residues within the active site of MKP-1/DUSP1 belong to the same IC. Q259 and I262 show a strong correlation. (**B**) KIM of MKP-1 where residues within the KIM of MKP-1 belong to 2 different ICs (K57 belongs to IC2, others to IC1). All residues except T50 in IC1 show a weaker correlation with K57 than with other residues in IC1. T50 shows a weaker correlation with R53—which is a residue in IC1—than with K57. (**C**) Positional correlation of positions within the active site of MKP-3. All residues within the active site of MKP-3/DUSP6 belong to the same IC. All residues show a similar level of covariance between each other. (**D**) Positional correlation of positions within the kinase interaction domain of MKP-3. Residues within the KIM of MKP-3/DUSP6 belong to 3 different ICs. G60, I61 and Q67belong to IC1. M62 and L63 belong to IC7. R64, R65, L66, K68, G69 belong to IC2. Residues in IC1 show stronger correlation with each other than with residues in other ICs and have weak correlation with other residues. All other residues show slightly stronger correlation with residues in their ICs, but also similar degree of correlation with other residues. (**E**) Positional correlation of positions within the active site of STYX. Residues within the active site of STYX belong to three different ICs. H119, N121, I124 and S125 belong to IC5. G120, A122 and R126 belong to IC1. G123 belongs to IC7. Positions belonging to the same IC show a higher degree of correlation compared to positions outside of their IC. (**F**) Positional correlation of positions within the active site of MK-STYX. Residues within the active site of MK-STYX/STYXL1 belong to 5 different ICs. F245 and I250 belong to IC7. S246 belongs to IC5. T247 belongs to IC2. Q248 belongs to IC8. G249, S251 and R252 belong to IC3. Positions belonging to the same IC show a higher degree of correlation compared to positions outside of their IC. (**G**) Positional correlation of positions within the kinase interaction domain of MK-STYX. Residues within the KIM of MK-STYX/STYXL1 belong to 6 different ICs. I48 belongs to IC5. T49, A50, R52 and K56 belong to IC1. L51 belong to IC9. V53 and K55 belong to IC2. K54 belongs to IC8. N57 belongs to IC6. Residues in IC1 have very a random degree of correlation. Residues in IC2 have strong correlation with each other. PyMOL Molecular Graphics System 4.6.0 (https://pymol.org/installers/PyMOL-2.3.3_0-Windows-x86_64.exe) was used to color the active sites and KIMs. Histograms were generated by MATLAB (https://www.mathworks.com/downloads/web_downloads/download_release?release=R2021a).

We next wanted to know how residues within the known motifs (active site and KIM) coevolved. We examined the covariation matrix for these regions in each of the proteins (Fig. 7). Most of positions within the active site and KIM support their strong inter-dependence, suggesting coevolution. Depending on the protein, these residues are sometimes in the same sector and sometimes split amongst related sectors. Residues within the active site of MKP-1 belongs to the same IC, and Q259 and I262 showed the strongest correlation between each other (Fig. 7A). All residues within the active site of MKP-3 belong to the IC and have similar covariance between them (Fig. 7C). Residues within the active site of STYX belong to three different ICs. IC1 consists of G120, A122, and R126; IC5 consists of H119, N121, I124 and S125, and IC 7 consists of G123 (Fig. 7E). Residues within the active site of MK-STYX belong to 5 different ICs. IC2 consists of residue T247; IC3 consists of G249, S251 and R252; IC5 consists of S246; IC7 consists of F245 and I250; and IC consists of Q248 (Fig. 7F). Positions belonging to the same IC show a higher degree of correlation compared to positions outside of the IC. The positional correlations within the kinase interaction domain (KIM) were also analyzed (Fig. 7B,D,G). The median covariation within the AS/KIM sites of MK-STYX are most like MKP-3.

The coevolutionary covariance of the residues in the active site of the active homologs supports the importance of the active site within MKPs, as well as PTPs in conserving the biological functions of these enzymes to regulate MAPK signaling. Although, the point mutations within the active motifs of the pseudophosphatases rendered them catalytically inactive, structural data presented here demonstrate that they do not affect the 3D structure for binding “substrates” (Fig. 6). It has been well established that some pseudophosphatases maintain their 3D fold^9,21^. The mutated G120 belongs to the same IC as the critical R126 (IC1) in STYX (Fig. 7). Furthermore, the DUSP of STYX resembles that of MKP-1, and STYX competes with MKP-1 for ERK binding^32^. The mutated S245 (IC5) and the critical R252 (IC3) in MK-STYX showed independent evolution (Fig. 7). These results suggest that the pseudophosphatases follow more divergent patterns of coevolution among residues of the active sites. In addition, it indicates another clear evolutionary distinction that MK-STYX was more divergent than STYX at the critical residues. The correlation, two sectors, and the structure of the active loop of MKP-1 and MK-STYX suggests that they may have similar biological functions. However, MK-STYX has not been reported to interact with ERKs^33^. This may be due to mutations within in the KIM domain that possibly prevent interactions with MAPKs, which dock to the KIM domain^17^. MK-STYX had more ICs in the KIM than MKP-1 and MKP-3 (Fig. 7), which also demonstrates the divergence of MK-STYX. Noteworthy, the consecutive arginines of MKP-1 and MKP-3, which are responsible for MAPK docking, were represented in the same IC in both proteins (IC1 in MKP-1, IC2 in MKP-3). However, the LRV of MK-STYX, which is in place of the consecutive arginines, belonged to three different ICs, which could further explain why MK-STYX fails to bind MAPKs from an evolutionary view (Fig. 7). Mutations within the KIM domain drastically change the structure of MK-STYX (Fig. 6). The SCA analysis revealed the evolutionary coupling and provide some insight to better understand the functions of these pseudophosphatases; however, more analysis beyond this study will be needed.

## Discussion

Phosphatases have long been characterized as merely ‘erasers’ in the phosphotyrosine-based signaling, simply serving as negative regulators of kinase signaling^34^. Following this analogy, pseudophosphatases have been further misconstrued as ‘dead erasers,’ serving no role or very little role as signaling molecules^3^. Fortunately, scientists have been working relentlessly to remove such misconceptions, providing evidence that phosphatases and pseudophosphatases are essential signaling molecules in numerous signaling pathways in their own right^4,21^. In particular, MK-STYX has been shown to regulate the mitochondrial-dependent apoptosis, promote neurite formation, and decrease the formation of stress granules, and STYX participates in the MAPK/ERK pathway, regulates the SCF-dependent ubiquitination activity, and is critical for spermatogenesis^19,31,32,35–38^. In addition, MK-STYX and STYX have been associated with oncogenesis, which further demonstrates the potential of targeting pseudophosphatases for cancer therapeutics^3,6,7,31^.

Computational tools have been important asset for investigating and understanding PTPs^10,11^. Bioinformatics identified MK-STYX as a DUSP; in particular, an inactive member of MKPs^9,12^. MK-STYX may be unique to vertebrates; homologues of MK-STYX have only been detected in phylum Chordata^12^. MK-STYX has been reported to be expressed in zebra fish, mice, and humans, but not insects, C. elegans or yeast^12^. Here, we provide a comprehensive genomic analysis of MK-STYX and STYX conservation throughout evolution.

Our analysis revealed two distinct patterns of their evolutionary conservation. Phylogeny of MK-STYX was mostly consistent with the source species, especially the class odontoceti and the class aves. STYX had the most consistency with the class odontoceti and strong consistency with the class aves and carnivores. However, the primates are separated in two groups by rodents and carnivores. Our data shows that MK-STYX had one of the highest protein distances of all DUSPs and CH2-domain-containing proteins, suggesting that it is rapidly changing and might have different functions among species. The rapid changing of MK-STYX among species suggests that MK-STYX may have different biological or signaling function throughout evolution. MK-STYX arose later in the phylogeny^39^, suggesting that its function is adaptable and/or dependent of environmental factors. This supports the initial unexpecting findings that MK-STYX does not interact with the expected binding partner MAPK^16,33,40^ and unpublished data). Furthermore, our recent computational mutagenesis findings demonstrate that MK-STYX does not maintains its three-dimensional fold within its KIM^40^. MK-STYX has a mutation in this motif, ablating the required consecutive arginine, for docking of MAPKs to this area^40^—suggesting that the rapid changing of MK-STYX changes its interacting partners, which could implicate it in many signaling pathways. It is well established that the evolutionary conservation of a structure/fold is important for substrate specificity throughout organisms. Although MK-STYX lacks the conserved fold at the KIM, MK-STYX maintains its fold in the DUSP domain^40^, allowing it to maintain its ability to bind phosphorylated residues such as its active homologs.

In contrast, the protein distances of STYX were smaller, ~ 82% of the DUSP-containing proteins, including MKP-1, an active homolog of MK-STYX. Thus, the sequences of STYX are highly conserved among species, suggesting it has essential roles in cell signaling. We also noticed a purifying selection imposed for the DUSP- and CH2-domain containing proteins. However, this selection pressure for MK-STYX is weaker than 80% of proteins in both groups. The selection pressure for STYX was opposite of MK-STYX; selection pressure was greater in ~ 80% of DUSPs. Furthermore, the selection pressure of STYX is equivalent to the strength of the selection pressure for MKP-3. The Ka/Ks ratios of the base-pair windows reveal that the CH2 domain of MK-STYX was under much weaker purifying selection pressure than those of MKP-1 and MKP-3, and the DUSP domain, including the active site, of MK-STYX was under weaker purifying selection pressure than those of STYX, MKP-1, and MKP-3. This strong purifying pressure of STYX, which is similar to the active MKP-1 and MKP-3 suggests that STYX has biological roles similar to these phosphatases. Indeed, STYX has a role in MAPK signaling cascades and competes with MAPKs^32,40^. The strong conservative selection pattern of STYX, MKP-1, and MKP-3 suggests that have biological similar roles, which has been reported^32^. Nevertheless, all four proteins share an evolutionary pattern at their N-terminal sequences that highlight spikes of positive selection pressure. It is important to note that while our method of determining Ka/Ks doesn’t account for recombination, several newer methods could be use such as coalescent simulation of intracodon recombination, inferring natural selection operating on conservation and radical substitution at single amino acid sites^41–43^.

Our SCA analysis also demonstrates that MK-STYX and STYX have very distinctive evolutionary patterns. MK-STYX was shown to correlate more with MKP-1, both forming complete separation of ICs and two sectors; whereas STYX and MKP-3 did not form complete separation of ICs and formed three sectors. However, the ICs at the active site and the KIM both formed a single sector for all MKPs, MK-STYX, MKP-1, and MKP-3 (Fig. 6)—demonstrating that the functional units of MK-STYX did not alter despite being catalytically inactive. Intriguingly, the KIMs and active sites of MK-STYX and MKP-3 both constitute one sector, whereas motifs of MKP-1 belong to two different sectors. This correlation between MK-STYX and MKP-3 suggests that MK-STYX, similar to MKP-3, may interact with cytoplasmic molecules that are non-MAPK proteins—beyond the reported non-MAPK partners such as G3BP-1 (Ras-GTPase activating protein SH3 domain binding protein-1)^19,36^ and PTPM1 (PTP localized to the mitochondrion 1)^35^. Future experiments are required to explore why the functional architecture of MK-STYX was closer to MKP-3 (Fig. 6). Unlike MKP-1 and MKP-3, MK-STYX active site and KIM domains are within the same sector– highlighting that this strong correlation may suggest coevolution. Intriguingly, the residue position correlation showed that residues within the active site of MK-STYX were within five ICs, whereas the active site for STYX were within three ICs (Figs. 6 and 7). Furthermore, residues within in the active site of MKP-1 and MKP-3 were in one IC, not, five and showed strong covariance. This demonstrates the divergence of MK-STYX from its active homolog at the amino acid level. Moreover, it may explain how MK-STYX elicits pleiotropic effects such as subcellular localization, turnover, various protein interactions, and regulation resulting in numerous cellular phenotypes^12^.

Regarding STYX, its FQQ motif distinctively interacts with the F-box protein FBXW7 and regulates the ubiquitination pathway^31^. The FQQ motif belonged to sector 2; however, the critical mutated G120C and R126 are within sector 1 (data not shown). Moreover, IC5 consists of the phenylalanine in the FQQ motif and four residues H119, N121, I124 and S125 in the active site all belong to IC5 (data not shown). Therefore, the function of the FQQ motif was clearly the result of an evolutionary path at the amino-acid level divergent from the canonical phosphatases and MK-STYX. However, it has been reported that the STYX-G120C mutant does not affect the formation of the STYX-FBXW7 complex in the nucleus. Thus, more studies are required to understand the insights behind the coevolution between the phenylalanine and the four residues in the active site^31^.

## Conclusion

Previous computational and structural evolutionary studies characterized the core structural conservation and conserved amino acids of specific domains of PTPs^10,11^. The current study provides an extensive genomic analysis on both MK-STYX and STYX, while highlighting a comparison among pseudophosphatases and two active phosphatases relative to their evolutionary conservation at multiple levels: protein sequence, coding sequence, and regional coding sequence. We also go beyond classic conservation to loop at evolutionary coupling within these proteins. Our dataset generated 68 DUSP-domain containing proteins and 36 CH2-domain-containing proteins, excluding entries that do not have a common gene symbol. Furthermore, these computational approaches show that MK-STYX and STYX have very distinctive evolutionary patterns, further confirming the independence and the uniqueness of each pseudophosphatase’s functional role as a signaling regulator. STYX is highly conserved and is under strong purifying pressure to resist change, which is important for a signaling molecule to have a role in well-established MAPK signaling pathway^32^. However, MK-STYX is under less purifying pressure and changes from organism to organism, such plasticity may be important for a molecule to have a role in numerous pathways. Whether under strong or weak purifying pressure, our studies show that pseudophosphatases are fascinating to explore, and are evolving to be important candidates for signaling pathways and diseases^3,4,6,44^. Recent reports extensively describe and classify the phosphatomes in human and other species^28,45^, which serve as a platform for more detailed genomic studies such as presented here. With the constantly increasing databases and accurate statistics models, the field is positioned to harness the combination of the power of bioinformatics, structural biology, and biochemistry to demonstrate that pseudophosphatases, rather than being “dead”, are ‘vigorously alive.’

## Methods

### DUSP- and CH2-domain-containing proteins selection

To obtain all proteins with either the DUSP or CH2 of MK-STYX, the keyword search for ‘MK-STYX’ was performed with PFAM, the database of protein families^46^. PFAM annotates the DUSP domain as ‘DSPc’, described as ‘dual specificity phosphatase, catalytic domain’ with the accession PF00782. The CH2 domain is annotated as ‘Rhodanese’, described as ‘rhodanese-like domain’ with the accession PF00581. To maintain consistency, this study refers to these two domains as ‘DUSP’ and ‘CH2’. For each of the two domains, the ‘domain organization’ option within the database provided all protein sequences containing that domain, which consists of reviewed annotated entries (UniProt/Swiss-Prot) and unreviewed automated entries (UniProtKB/TrEMBL). These sequences downloaded with their UniProt Knowledgebase (UniProtKB) identifiers. All identifiers were searched with UniProtKB through its Retrieve/ID mapping function, with options set as ‘from UniProtKB AC/ID to UniProtKB.’ This search resulted in 25,896 active protein entries for the DUSP domain and 60,764 active protein entries for the CH2 domain. All results were downloaded as text format, which includes the largest extent of information relevant to this project such as protein sequences, gene names, organisms, and mapped identifiers to other databases. Text files were parsed and converted to CSV (comma-separated value) files by Python scripts for the convenience of data processing.

### Obtaining gene symbols

Gene symbols (e.g., *STYXL1, STYX, DUSP1*) were required for all entries downloaded from PFAM. Because most entries have gene names that are organism- or locus-specific (e.g., *loc101349488*, *vigan_02195500*, *F54D1.6*), efforts were made to assigned gene symbols to all entries. To try to obtain gene and protein descriptions of all protein entries, a search using the gene name, Gene ID, RefSeq Protein accession, and Ensembl Gene ID were programmatically searched performed on either NCBI or Ensembl. An algorithm was used to parse those description and assign a gene symbol to each entry; entries without valid gene symbols were removed. This study analyzed 92 gene symbols, among which 68 genes have the DUSP domain and 36 genes have the CH2 domain.

These 92 gene symbols were searched through NCBI Gene and UniprotKB to obtain orthologs and/or paralogs for all genes and their protein sequences. Entries from PFAM, NCBI Gene, and UniprotKB were merged based on gene symbols. The merged entries of MK-STYX and STYX, resulted in a list of 433 species that express either MK-STYX, STYX, or both. Genes entries with species that were not in the list were removed. When multiple entries of one gene had the same organism, only the longest protein sequence was kept.

### Obtaining sequences

The protein sequences of all entries in the dataset were downloaded from UniProtKB. All entries with protein sequences that did not start with methionine were removed. The mRNA coding sequences (CDS) of proteins were retrieved from the NCBI, EMBL-EBI, or Ensembl database. To automate the search and retrieval processes through the NCBI Entrez system (db = nucleotide), the EBI Search RESTful API (dbName = ena_coding), and the Ensembl REST API (type = cds), scripts were written in Python. When the coding sequence of an entry was available through multiple databases, the prioritization order was NCBI, EMBL-EBI, and Ensembl. All entries with coding sequences that did not start with 'ATG' were removed.

### Sequence alignments

Protein sequences were aligned through MEGA-X using the MUSCLE alignment in MEGA-X, Molecular Evolutionary Genetics Analysis across computing platforms^47,48^. The gap open cost was set at -10.0, a gap extended cost of—0.10, and the hydrophobicity cost of 1.20, maximum iterations set to 16, and genetic code as standard. The cluster method *UPGMA* was chosen, with a minimum diagonal length (lambda) of 24. Distance matrices were calculated from six different models: equal input, Jones–Taylor–Thornton (JTT), number of differences, p-distance, Poisson, and Dayhoff^48–50^.

Nucleotide sequences were aligned through MEGA-X using the MUSCLE algorithm with a gap opening penalty of -15.00 and gap extension penalty of -6.70, a hydrophobicity cost of 1.20, the maximum iterations set to 16, the genetic code as standard, the cluster method for all iterations as UPGMA, and the a diag length (lamda) of 24^47,48^.

### Evolutionary trees

To construct evolutionary trees of organisms expressing MK-STYX, STYX, and for all organisms within our dataset, Newick tree files were created using the free online software phyloT: a tree generator that is based on NCBI taxonomy. Constructed tree files were imported into the Interactive Tree of Life (iTOL)^51,52^ and exported as PNF files for analysis. The phylogenetic tree respective to all organisms was annotated using iTOL by coloring clades. The trees for STYX and MK-STYX were annotated using the ETE3 Toolkit with colored nodes indicating the species-species distance value with respect to the equal input model distance matrix of each protein.

### Protein distance analysis

To compare proteins in similar species, all species-species pairs were put into 4 bins bordered by 0.247241104, 0.42821296, and 0.699670069. The borders were determined by the most distinguishable troughs observed from the frequency distribution of the mean protein distances (by the Equal Input model) of all species pairs. In each bin, for every protein, all available pairwise distances were aggregated to produce a mean distance. The rank of that protein was determined by the percentile of its mean distance. The results were analyzed separately for both DUSP-containing proteins and CH2-containing proteins and plotted as cumulative probability histograms for all six models. A lower percentile suggests that a protein has higher distances among species and thus is changing rapidly. In contrast, a higher rank demonstrates that a protein has high sequence conservation.

### Selection pressure (Ka/Ks) analysis for complete coding sequences

The nonsynonymous mutation rates (Ka) and synonymous mutation rates (Ks) were calculated on every sequence pair by MEGA-X using the Nei-Gojobori with a Jukes-Cantor model^53^. Other settings included the no variance estimation method and pairwise deletion for gaps/missing data treatment. The Ka and Ks matrices of all genes were imported to Jupyter Notebook for data processing. All ‘?’ in the matrices were removed, and all values equal to zero were replaced with 1e-15 to avoid zero division error. The log_10_(Ka/Ks) values for all organism pairs of every gene in the dataset were calculated. Analysis on selection pressure was also conducted within the four bins. For each bin, all pairwise Ka, Ks, and log_10_(Ka/Ks) values were aggregated to produce the corresponding median values for every gene. The ranks of that gene were determined by the percentile of these median values.

### Selection pressure (Ka/Ks) analysis for coding-sequence windows

To obtain the 99 base pair windows, shifting each window by 9 base pairs, a python script was used to create a text file containing the 99mers of each organism from their full alignments of four genes (MKP-1, MKP-3, STYX, and MK-STYX). An embedded AutoHotkey script was then employed to automate the analysis of the Ka and Ks of each sequence window in MEGA-X with the same settings described above. The regional log_10_(Ka/Ks) was calculated similarly for all windows of the four genes of interest (MK-STYX, STYX, DUSP1, and DUSP6). To determine the regional selection patterns of genes, the median values were taken of the log_10_(Ka/Ks) ratios of all 99-bp ‘windows’ 9 bp apart along a coding sequence alignment and plotted in an ‘iceberg’ graph for MK-STYX, STYX, MKP-1, and MKP-3. The domains of each protein in the iceberg graphs were determined by identifying the first window that starts at the first nucleotide of a particular coding region of a domain, and the last window that ends at the last nucleotide of that coding region. The motifs in the graphs were determined by identifying the first window that ends with the complete motif sequence and the last window that starts with the complete motif sequence.

### Statistical coupling analysis (SCA)

To determine the structural and biochemical evolutionary meanings of MK-STYX, statistical coupled analysis (SCA) was performed to analyze the constraints of amino acids within it. Two inputs, multi-sequence alignment (MSA) and protein data bank (PDB) structure were imported in SCA MATLAB implementation, which allows SCA analysis on any protein. In this study, 309 sequences were collected for MKP-1/DUSP1; 364 sequences were collected for MKP-3/DUSP6; 419 sequences were collected for STYX; 347 sequences were collected for STYXL1/MK-STYX. Iterative Threading ASSEmbly Refinement (I-TASSER)^54^ was used to predict the structure of MKP-1, MKP-3, STYX, and STYXL1/MK-STYX, which have no reported full length crystal structure in PDB. PDB structures for MKP-3 and STYX are 1MKP and 2R0B, respectively. For each I-TASSER predicted structure, three confidence measures were generated, the TM-score, the root-mean-square deviation (RMSD) and the C-score. The TM- score measures topological similarity between a protein structure generated by I-TASSER and a pre-existing structure from PDB. I-TASSER automatically ranks the top five predicted structures by their overall residue-level local accuracy from highest to the lowest. The residue-level local accuracy is defined as the distance deviation, which is measured in Angstroms, between the positions of residues in the prediction and that in the native structures^55^. The best fitted model which had the highest overall residue-level local accuracy was chosen^55^. Therefore, the first model was chosen for all four proteins in this study.

MSA was subject to the following pre-processing steps: (1) sequences with too many gaps were removed to prevent too much noise in downstream analysis; (2) positions where more than a specified fraction (default cutoff of 0.4)^26^ of sequences in the MSA have a gap were truncated; (3) sequences with more than a certain fraction (default cutoff of 0.2)^26^ of gaps were removed; (4) both the truncated MSA and the pdf file were aligned to create an applied type sequence (ATS) array, which allowed residues in every given sequence to be mapped to the sequences in the pdf structure. Alternatively, the similarities between every pair of sequences were computed and data presented as a heatmap, to determine whether too much "noise" (e.g. different protein sequences) were present in MSA.

### First-order statistics—positional correlation

To determine the evolution of individual residues the degree of conservation was calculated. For a large and diverse MSA with at least 100 effective sequences, the evolutionary conservation of each amino acid residue was measured by the Kullback–Leibler relative entropy as the following:

$${D}_{i}^{a}={f}_{i}^{a} ln\frac{{f}_{i}^{a}}{{q}^{a}}+\left(1- {f}_{i}^{a}\right) ln\frac{1- {f}_{i}^{a} }{1- {q}^{a}}$$

where ${f}_{i}^{a}$ is the observed frequency of amino acid $a$ at position $i$ in the alignment and ${q}^{a}$ is the background expectation^26^.

### Second-order statistics—conserved correlations

The amino acid residues within a protein cooperate with each other in folding and function. To investigate the cooperativity and to determine coevolution between positions in a protein, the position-specific conservation of individual amino acid residues must be extended to pairwise conservation^26^. The conservation of a certain pair of amino acids (a, b) at positions (i, j) are calculated to be the difference between their joint frequency ${f}_{ij}^{ab}$ and that expected in the absence of correlation ${f}_{i}^{a}{f}_{j}^{b}$. A covariance matrix can be defined for all such pairwise conservation in a given protein as the following:

$${C}_{ij}^{ab}= {f}_{ij}^{ab}- {f}_{i}^{a}{f}_{j}^{b}$$

It is judged by the degree of conservation of the underlying amino acids:

$${\tilde{C }}_{ij}^{ab}= {\phi }_{i}^{a}{\phi }_{j}^{b}{C}_{ij}^{ab}\,,\text{in which}\,{\phi }_{i}^{a}= \frac{\partial {D}_{i}^{a}}{\partial {f}_{i}^{a}}=\text{ln}\left[\frac{{f}_{i}^{a}(1- {q}^{a}) }{\left(1- {f}_{i}^{a}\right){q}^{a}}\right]$$

By computing the “Frobenius norm” of the 20 × 20 matrix of ${\tilde{C }}_{ij}^{ab}$ for every pair of (ij)

$${\tilde{C }}_{ij}= \sqrt{\sum_{a,b}{({\tilde{C }}_{ij}^{ab})}^{2}}$$

is derived^26^. The positional correlation matrix ${\tilde{C }}_{ij}$ was transformed to compute eigenvalues and eigenvectors. The top eigenvectors were further decomposed into high-level statistical coupling units using independent component analysis (ICA).

To analyze the positional correlations and to carry out ICA, the numbers of ICs assigned to a specific protein was determined. Spectral decomposition was used for the transformation of the positional correlation matrix into ICs. Per the decomposition, the ${\tilde{C }}_{ij}$ matrix is written as the following:

$$\tilde{C} = \tilde{V}\mathop \Delta \limits^{\sim } \widetilde{{V^{T} }}$$

where $\tilde{V }$ is an L × L diagonal matrix of eigenvalues (ranked by magnitude) and $\stackrel{\sim }{\Delta }$ is an L × L matrix whose columns contain the associated eigenvectors^26^. Ten randomized trials were performed on each of the four proteins to determine the true positional correlations, and to filter away the spurious correlations expected due to finite sampling in the alignment^26^. A high number of trials such as 100 did not yield a different result, but lead to significantly longer computing time; therefore, N = 10 was chosen. A cutoff was drawn on the spectral decomposition figure to determine the number of significant eigenmodes of ${\tilde{C }}_{ij}, \text{which\,provides\,the}$ of ICs. This variable is the kmax or k*^26^. The cutoff for significant eigenvalues is ${\lambda }_{2}^{rand}+2\sigma$, the second random eigenvalue plus two standard deviations computed over N randomization trials (Rivoire, 2016). The ${\lambda }_{2}^{rand}$ was calculated by taking the mean of all second random eigenvalue over ten trials. The standard deviation was calculated to be the average standard deviation over ten trials. The number of significant eigenvalues is the number of bars to the right of the cutoff.

ICA is the extension of the spectral decomposition and the precursor of sector definition. ICA is able to deduce a matrix W that transforms the kmax/k* top eigenmodes into kmax/k* maximally independent components^26^:

$${\tilde{V }}_{1\dots k}^{p}=W{\tilde{V }}_{1\dots k}$$

To determine whether ICs were successfully separated from each other, 3-D scatter plot for the top three ICs for each of the three proteins were plotted. Strongly correlated positions appear nearby, while weakly correlated positions are far apart. The positions that contribute substantially to any of the top three ICs should appear far away from the origin, whereas the positions that do not contribute substantially to any of the top three ICs should cluster near the origin^26^. ICA assumes the existence of quasi-independent groups. Ideally, positions contributing to the top three ICs are expected to be at a distance from the origin and are along the three axes to form an orthogonal shape^26^.

Generally, each IC arises from one of the following two possibilities: (1) a truly independent sector with a distinctive function and (2) a purely phylogenetic clustering of sequences from the decomposition of one sector^26^. To distinguish between the two possibilities each IC was placed into an empirical statistical distribution, the positions that contributed to the top 5% of the cumulative density function was determined^26^. T-distribution, which has been reported to work well for most cases^26^, was used for all ICs of all proteins in this study. All the selected positions will be arranged in a sub-matrix. The sub-matrix of all the selected positions from all ICs was transformed into a heatmap. A hot color such as red, orange, and yellow represent a shorter distance between the two residues; the cold color such as blue and green represent a longer distance between the two residues.

### Sector determination

Sectors are defined as the optimal representation of distinctive functions in a protein. This study defines a sector as the grouping of ICs with no significant statistical difference. In addition, an ANOVA test was performed for every pair of ICs on the heatmap to compute a matrix of p-values, with a cutoff of 0.10. All positions in a protein cooperate to function, and a too small p-value results in all ICs merging into one giant sector; therefore, p = 0.10 was chosen. ICs with p-values above the cutoff have no statistically significant difference and were grouped together into a sector. For all four proteins, the positional correlation and the positional conservation regarding other residues within the active site were computed for all the residues within the active site. In addition, for MKP-1/DUSP1, MKP-3/DUSP6 and STYXL1/MK-STYX, the positional correlation and the positional conservation were also computed for all the residues within the kinase interaction motif (KIM).

## Supplementary Information

Supplementary Figure Legends.

Supplementary Information.

Supplementary Information.

Supplementary Figure S1.

Supplementary Figure S2.

Supplementary Figure S3.

Supplementary Figure S4.

Supplementary Figure S5.

## Data Availability

The software scripts written for use with python and MATLAB are available as source code in the supplement. The AutoHotKey scripts are also available in the supplement.

## Abbreviations

API: Application programming interface
CD: Cluster of designation
CDC: Cell-division cycle
CDC#: Cell-division cycle phosphatase with an assigned number
CDK: Cyclin-dependent kinase complex
CDS: Coding sequences
CH2: Cell division cycle 25 phosphatase homology 2
CSV: Comma-separated values
DNA: Deoxyribonucleic acid
DUSP: Dual-specificity phosphatase or the dual-specificity phosphatase domain
EMBL-EBI: European Molecular Biology Laboratory – European Bioinformatics Institute
ERK: Extracellular signal-regulated kinases ½
ETE 3: Environment for tree exploration
G3BP-1: Ras-GTPase activating protein SH3 domain binding protein-1
I-TASSER: Iterative Threading ASSEmbly Refinement
iTOL: Interactive Tree Of Life
KIM: Kinase interaction motif
Ka: Number of nonsynonymous substitutions per nonsynonymous site
Ks: Number of synonymous substitutions per synonymous site
MAPK: Mitogen-activated protein kinase
MEGA: Molecular evolutionary genetics analysis
MEK: Mitogen-activated protein kinase kinase
MKP#: MAP kinase phosphatase with an assigned number
MK-STYX: Mitogen-activated protein kinase phosphoserine/threonine/tyrosine-binding protein
mRNA: Messenger ribonucleic acid
MUSCLE: Multiple sequence comparison by log-expectation
NCBI: National center for biotechnology information
pERK: Phospho-ERK
pSer: Phospho-serine
pThr: Phospho-threonine
pTyr: Phospho-tyrosine
PTP: Protein tyrosine phosphatase
PTPM1: PTP localized to the mitochondrion
REST: Representational state transfer
SCF: SKP/Cullin1/F-box complex
STYX: Phosphoserine/threonine/tyrosine-interacting protein
STYXL1: STYX-like-1
UniProtKB: Universal protein knowledge base

## Code availability

Available in supplement.
