# Supplementary material for: Evolutionary genomic relationships and coupling in MK-STYX and STYX pseudophosphatases
Source: Sci Rep. 2022 Mar 9;12:4139. doi: 10.1038/s41598-022-07943-5 (PMC8907265; doi:10.1038/s41598-022-07943-5)
Supplement: Supplementary file 1 — Supplementary Figure Legends. [file 41598_2022_7943_MOESM1_ESM.docx]

**Supplemental Figure Legends**

**Supplemental Figure 1. Phylogenetic Trees for all organisms, STYX, and MK-STYX**

(A)Phylogenetic tree for all organisms in our dataset. (B)Phylogenetic tree for STYX with unincluded organisms: Physeter macrocephalus, and Cryptococcus gattii. (C)Phylogenetic tree for MK-STYX with not included organisms: Physeter macrocephalus, and Lingula unguis

Organisms that are not included in a tree were unable to be placed in a tree based on phylogeny. Phylogenetic trees were generated by phyloT (<https://phylot.biobyte.de/index.cgi>) and analyzed and displayed with iTOL ([version 1.0](file:///Users/SDHinton/Desktop/Scientific_Rpeorts_December_23_Amendments/Scientific_Rpeorts_December_23_Amendments/version%201.0)) <https://itol.embl.de/> and environment for tree exploration (ETE3) (<http://etetoolkit.org/>).

**Supplemental Figure 2. Frequency distribution of the mean distances of all species-species pairs.**

All protein distances between any two species were aggregated to produce the mean distance of that species-species pair. The figure shows the number of species-species pair within each distance interval. Vertical dash lines represent the three borders determined by the distribution that separate the four bins of all species-species comparisons.

**Supplemental Figure 3.** **SCA weighted positional correlations**. (A, C, E, G) Positional correlation covariance matrix of MKP-1, MKP-3, STYX and MK-STYX, ordered by the primary structure (i.e., first amino aide is top left corner, last is bottom right). A higher value of covariance is presented by a warm color, implying a higher degree of coevolution between each pair of positions. (B, D, F, H) The same positional correlation covariance matrix after hierarchical clustering and sorting by SCA weights. The dendrograms further illustrate the clustering of positions.

**Supplemental Figure 4. Spectral decomposition.** (A)The eigenspectrum of the covariance matrix (in blue bars) for MKP-1/DUSP1; (B) MKP-3/DUSP6; **(C)** STYX; (D) and MK-STYX/STYXL1. The red curve is the randomly expected eigenvalue distribution for each protein over 10 randomization trials. The cutoff is drawn at x = (second eigenvalue + 2 standard deviation) over 10 randomization trials. These eigenspectrums provide a statistical basis for defining the top kmax/k* significant eigenmodes, which are later defined as ICs. The results indicate that kmax/k* = 7 for (A) MKP-1/DUSP1; (B) MKP-3/DUSP6; (C) STYX; and (D) kmax/k* = 9 for MK-STYX/STYXL1. Spectral decomposition diagrams were generated by MATLAB (<https://www.mathworks.com/downloads/web_downloads/download_release?release=R2021a>) and the layout was designed in Microsoft PowerPoint.

**Supplemental Figure 5. ICA 3-D scatter plot.** In general, independent component analysis (ICA) tends to optimizes the independence of all ICs by putting them on orthogonal axes. Due to the limitation of 3-D representations, only the first 3 ICs for each protein are shown. The ordering of ICs has does not have any statistical significance. **A-D**, In this study, the top 5% positions of the cumulative density function (CDF) in each of the first 3 ICs are colored differently to distinguish from each other. The top 5% positions of IC1, IC2 and IC3 are colored in red, green and yellow, respectively. (A)The IC scatter plot of MKP-1/DUSP1 shows a clear separation of the first 3 ICs on the orthogonal axes. (B)The IC scatter plot of MKP-3/DUSP6 shows a clear separation between IC3 and the other two ICs. However, IC1 and IC2 do not seem to be independent from each other. It is a sign that IC1 and IC2 may belong to the same sector. (C)The IC scatter plot of STYX shows a clear separation between IC1 and the other two ICs. However, IC2 and IC3 do not seem to be independent from each other. It is a sign that IC2 and IC3 may belong to the same sector. (D) The IC scatter plot of MK-STYX/STYXL1 shows a clear separation of the first 3 ICs on the orthogonal. Scatter plots were generated by MATLAB (<https://www.mathworks.com/downloads/web_downloads/download_release?release=R2021a>) and the layout was designed in Microsoft PowerPoint.
