# Supplementary material for: Evolutionary genomic relationships and coupling in MK-STYX and STYX pseudophosphatases
Source: Sci Rep. 2022 Mar 9;12:4139. doi: 10.1038/s41598-022-07943-5 (PMC8907265; doi:10.1038/s41598-022-07943-5)
Supplement: Supplementary file 2 — Supplementary Information. [file 41598_2022_7943_MOESM2_ESM.pdf]

CH2 Aligned nucleotide:

Folder containing the nucleotide alignments in fasta format for all genes containing a CH2/rhodanese-like domain in our dataset.

CH2 Aligned protein:

Folder containing the amino acid alignments in fasta format for all genes containing a CH2/rhodanese-like domain in our dataset.

DSP Aligned nucleotide:

Folder containing the nucleotide alignments in fasta format for all genes containing a DSP/dual specificity phosphatase domain in our dataset.

DSP Aligned protein:

Folder containing the amino acid alignments in fasta format for all genes containing a DSP/dual specificity phosphatase domain in our dataset.

145_species_list.docx:

List containing the 145 organisms represented in the phylogenetic tree in Figure 1 A and B

145_species_phylogenetic_tree.docx:

Newick tree string for the phylogenetic tree in Figure 1 A and B

alignmentreader.py:

Python file with embedded AutoHotKey to automate the ka and ks analysis for the 99 base pair windows of our 4 main genes of interest that is represented in Figure 4

alignmentwindow.exe:

AutoHotKey script used to automate ks analysis for the 99 base pair windows of our 4 main genes of interest that is represented in Figure 4

alignmentwindowka.exe:

AutoHotKey script used to automate ka analysis for the 99 base pair windows of our 4 main genes of interest that is represented in Figure 4

All_organisms(list).docx:

List of every organism included in the phylogenetic tree in Supplemental 1 A

All_organisms_phylogenetic_tree.docx:

Newick string file for the phylogenetic tree represented by Supplemental 1 A

MKSTYX_phylogenetic_tree.docx:

Newick string file for the phylogenetic tree represented by Supplemental 1 C

PhyloTree.ipynb:

Jupyter Notebook used to automate phylogenetic tree annotations. Used to color nodes of the trees seen in Figure 1 A and B

styx_organisms(list).docx:

List of every organism included in the phylogenetic tree in Supplemental 1 B

STYX_phylogenetic_tree.docx:

Newick string file for the phylogenetic tree represented by Supplemental 1 B

styxl1_organisms(list).docx:

List of every organism included in the phylogenetic tree in Supplemental 1 C
