# Supplementary material for: Evolutionary genomic relationships and coupling in MK-STYX and STYX pseudophosphatases
Source: Sci Rep. 2022 Mar 9;12:4139. doi: 10.1038/s41598-022-07943-5 (PMC8907265; doi:10.1038/s41598-022-07943-5)
Supplement: Supplementary file 3 — Supplementary Information. [file 41598_2022_7943_MOESM3_ESM.pdf]

**All the codes should be opened by MATLAB. The version of MATLAB for this implementation is R2021a.**

STYXL1_v3.mlx

The main code for this implementation. The comments in the code should clearly indicate the locations of all inputs and outputs, along with the purpose of each step. This is the only file in this toolbox that is executable. All other files are either an input or a function in this main code.

**Inputs**

MKP-1_model1.pdb

This is the I-TASSER predicted structure for MKP-1.

MKP-3_model1.pdb

This is the I-TASSER predicted structure for MKP-3.

STYX_model1.pdb

This is the I-TASSER predicted structure for STYX.

STYXL1_model1.pdb

This is the I-TASSER predicted structure for MK-STYX.

dusp1.fasta

This is the multi sequence alignment for MKP-1.

dusp6.fasta

This is the multi sequence alignment for MKP-3.

styx.fasta

This is the multi sequence alignment for STYX.

styxl1.fasta

This is the multi sequence alignment for MK-STYX.

**Sca5**

basic_ica.m

Basic ICA algorithm which takes 3 inputs - L*M input matrix where L = # features and M = # samples, learning rate / relaxation parameter, number of iterations, and gives 2 outputs - unmixing matrix, record of incremental changes during the iterations.

cons.m

Computes the conservation of amino acids at positions in a multiple sequence alignment.

Entropy.m

Necessary function for cons.m.

Calculates Gibbs-Shannon Relative entropy D(f|q) if a is not specified or a=1, or Renyi (relative) entropy with parameter a otherwise.

MSAsearch.m

Makes pairwise alignments between a query sequence (from the pdb file and chain ID) and every sequence in an MSA (alignment), finds the tophit sequence, and then attempts to make a residue number list (ats) that relates alignment numbering to structure numbering.

sca5.m

Carries out statistical coupling analysis for a protein family given a multiple sequence alignment.

binrep.m

Necessary function for sca5.m.

Converts a standard ASCII multiple sequence alignment (M sequences x L positions) to a M x L x 20 3D binary tensor (X3d) representation.

weight_aln.m

Necessary function for sca5.m.

Takes in the binary tensor representation of a multiple sequnece alignment (x3d, generated by binrep.m), and outputs the weighted alignment tensor (wX) and the weight matrix (W).

DerivEntropy.m

Necessary function for sca5.m.

Derivative of relative entropy dD(f||q)/df.

project_aln.m

Necessary function for sca5.m.

Computes the projected weighted alignment (pwX_wf) given the alignment in ascii format (algn, to compute the frequencies), the weighted 3-D alignment tensor (wX, generated by weight_aln.m), and the weight matrix (W, generated by weight_aln.m).

eigenvect.m

Necessary function for sca5.m.

Returns all (or the only the first kmax) principal eigenvectors, w(:,1), w(:,2), ..., and their corresponding eigenvalues.

SCAcluster.m

Two dimensional hierarchical clustering of SCA correlation matrix using city-block distance metric and compete linkage.

scacursor.m

Function for easy interrogation of SCA correlation matrix data, and for extraction of cluster composition.

sim_seq.m

Returns matrices of correlation and similarity between sequences

lett2num.m

Necessary function for sim_seq.m.

Translates an alignment from a representation where the 20 natural amino acids are represented by letters to a representation where they are represented by the numbers 1,...,20. Any symbol not corresponding to an amino acid is represented by 0.

spectral_decomp.m

Computes the spectral (eigenvalue) decomposition of the SCA correlation matrices in the SCA structure file (in, generated by sca5.m), and carries out N_samples trials of such decomposition for randomized alignments.
