# Supplementary figures and images for: Evolutionary genomic relationships and coupling in MK-STYX and STYX pseudophosphatases
Source: Sci Rep. 2022 Mar 9;12:4139. doi: 10.1038/s41598-022-07943-5 (PMC8907265; doi:10.1038/s41598-022-07943-5)

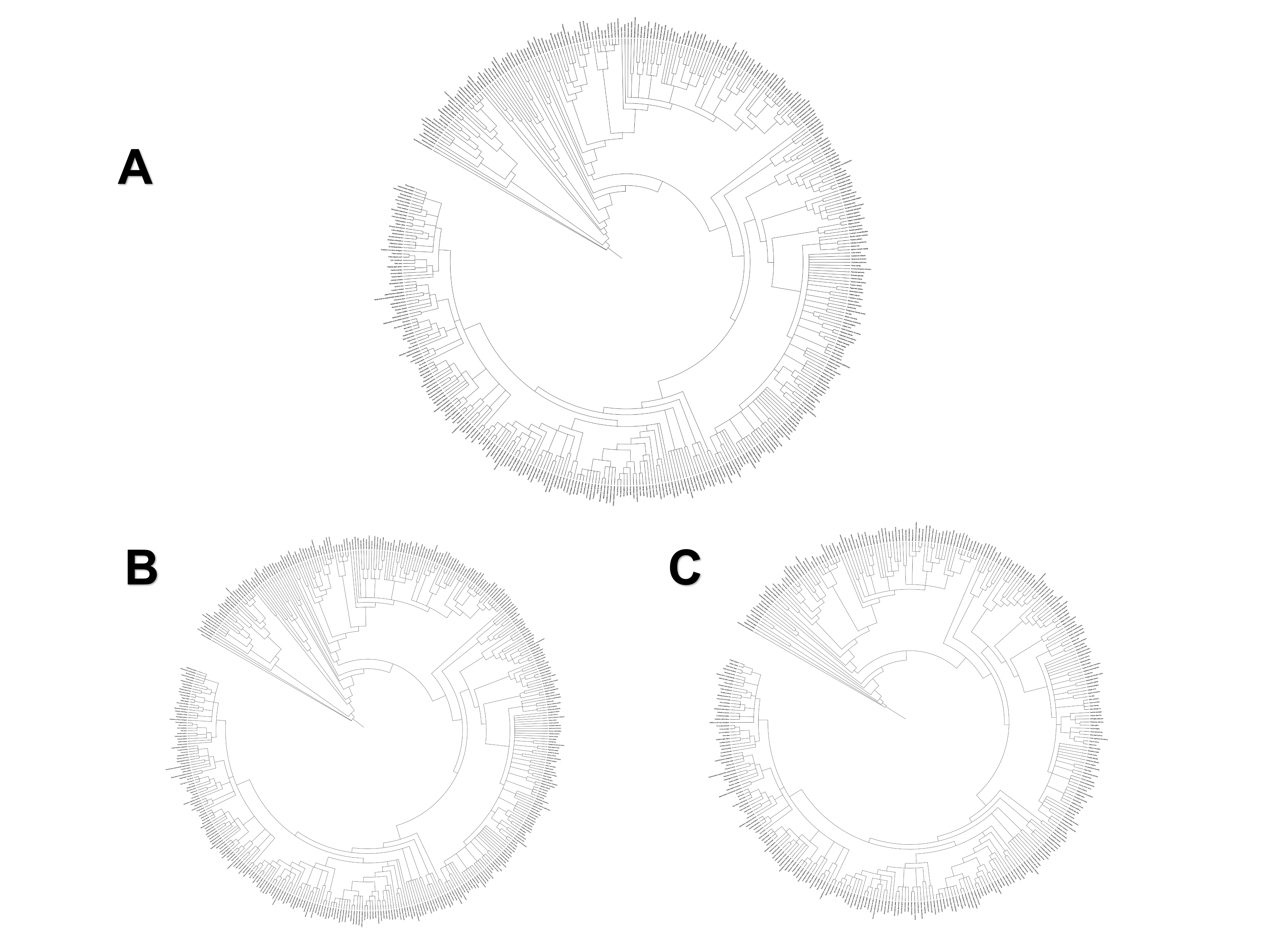

Supplement: Supplementary file 4 — Supplementary Figure S1. [file 41598_2022_7943_MOESM4_ESM.tif]

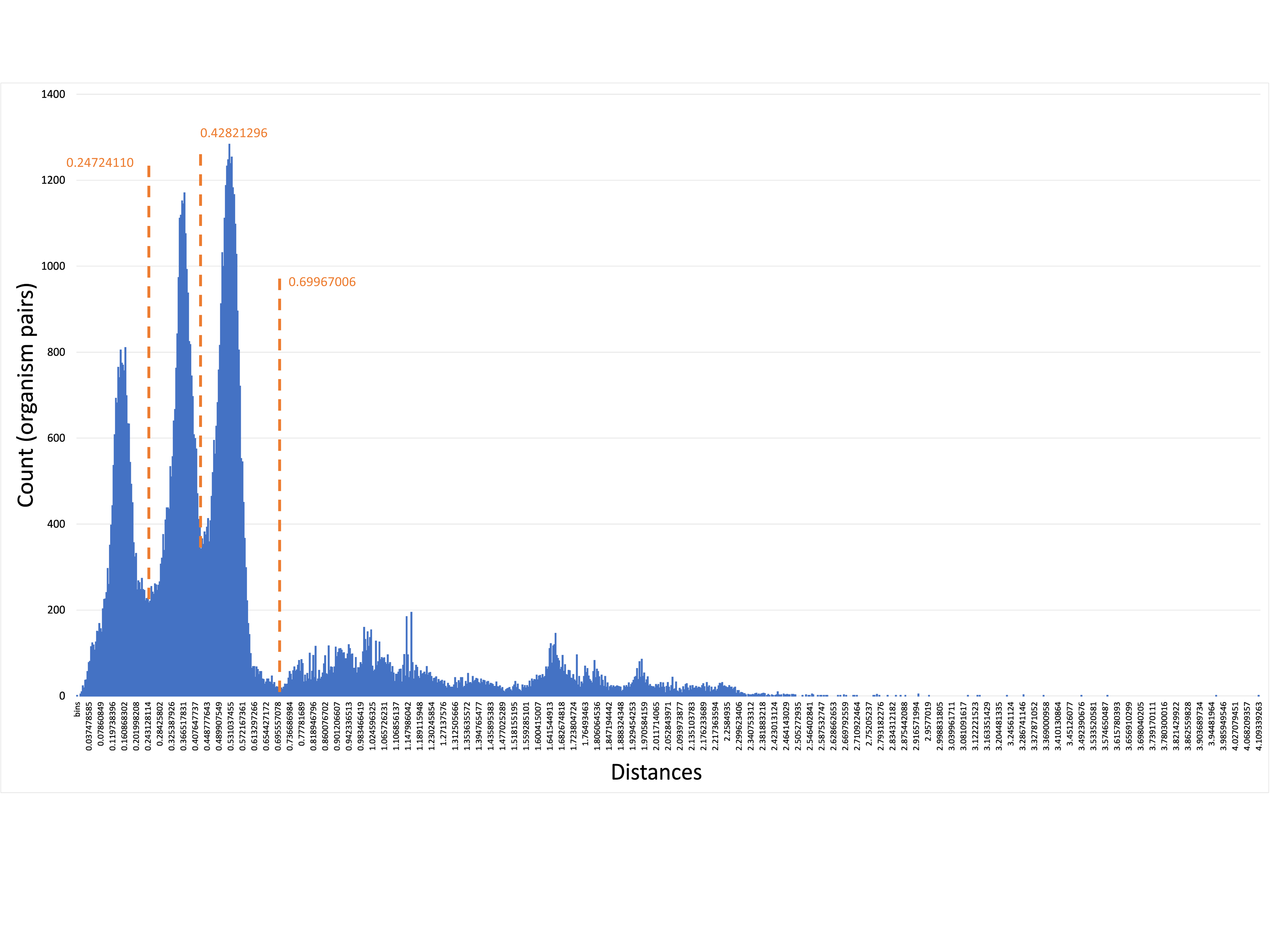

Supplement: Supplementary file 5 — Supplementary Figure S2. [file 41598_2022_7943_MOESM5_ESM.tif]

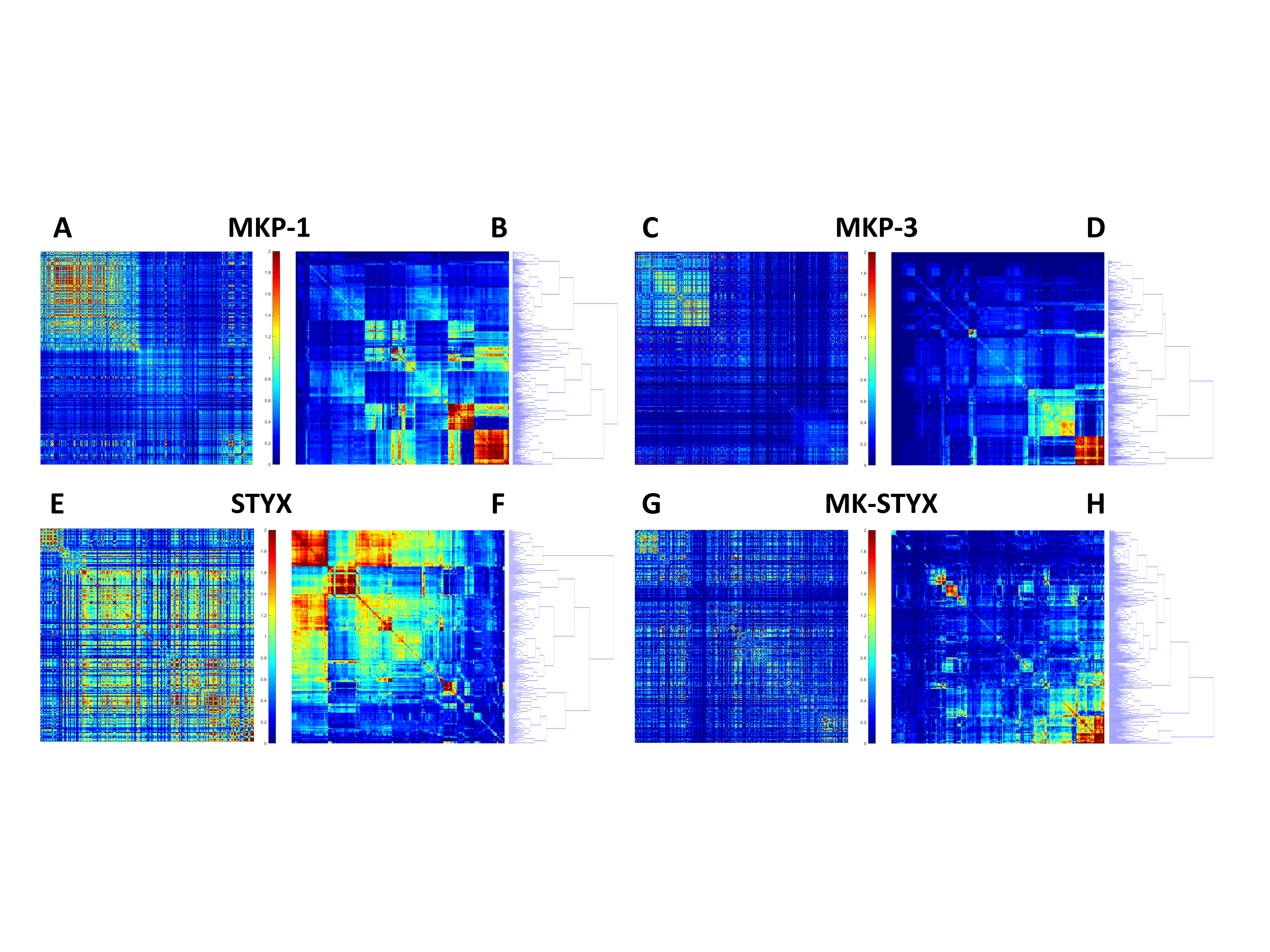

Supplement: Supplementary file 6 — Supplementary Figure S3. [file 41598_2022_7943_MOESM6_ESM.tif]

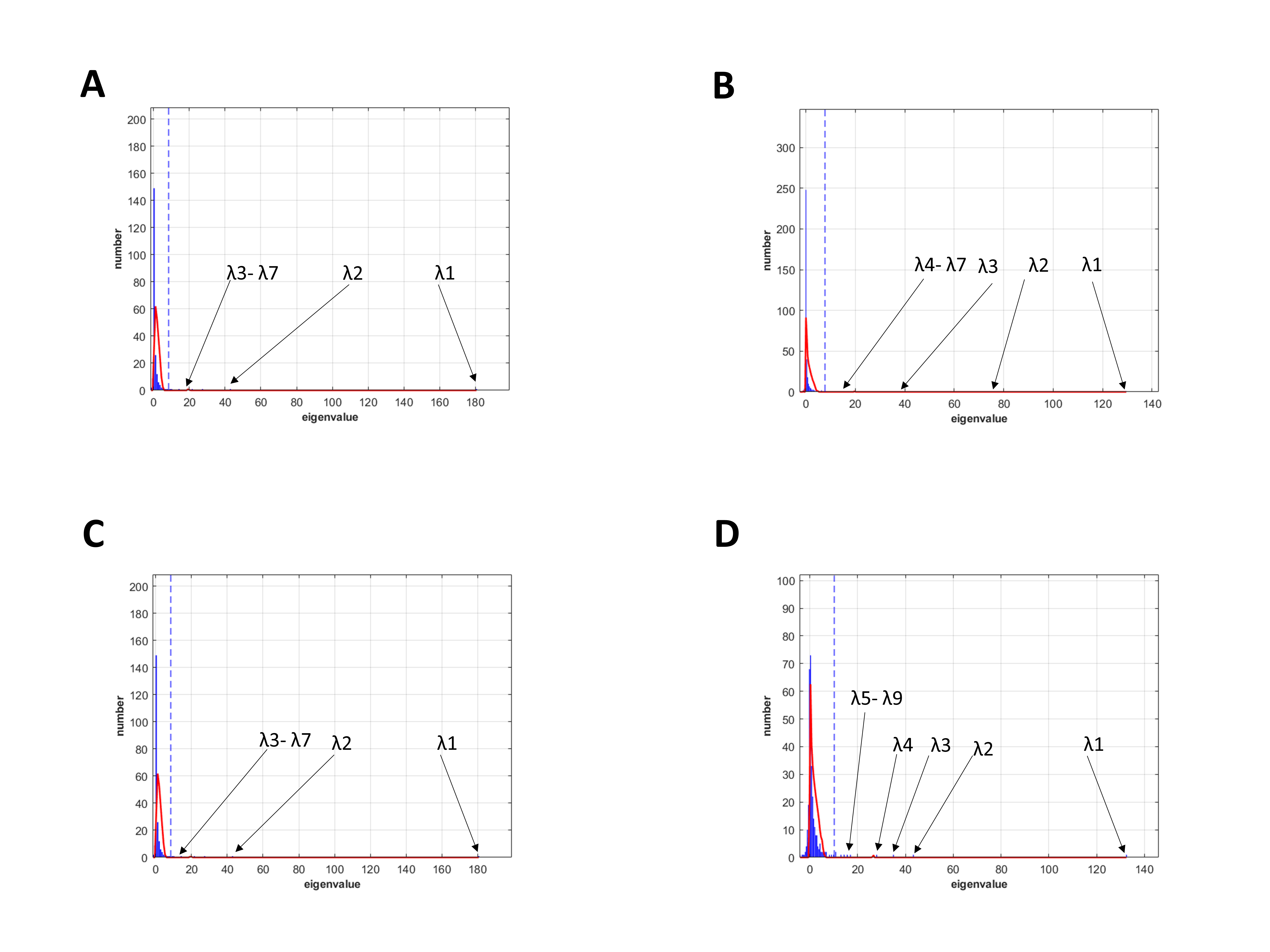

Supplement: Supplementary file 7 — Supplementary Figure S4. [file 41598_2022_7943_MOESM7_ESM.tif]

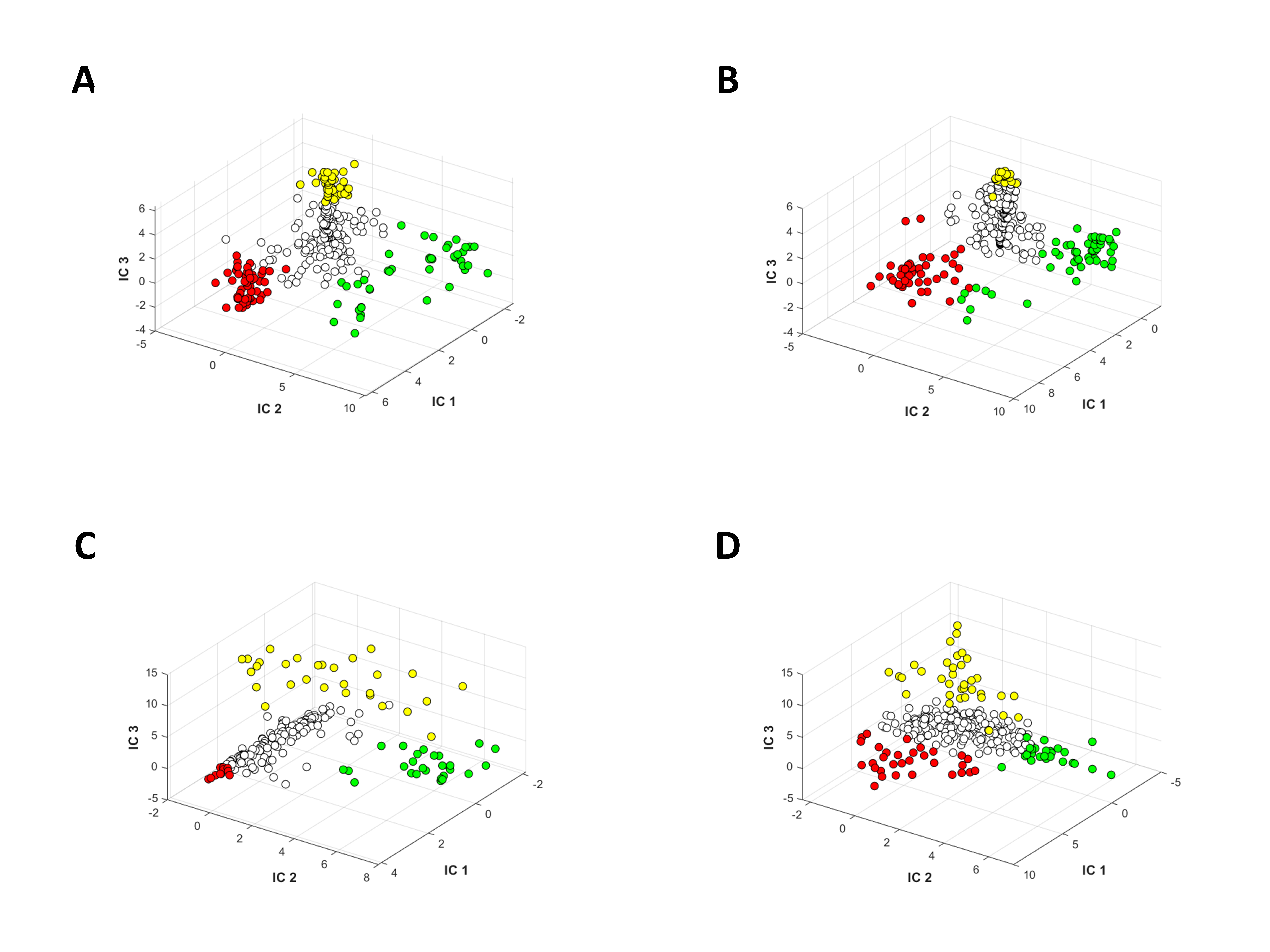

Supplement: Supplementary file 8 — Supplementary Figure S5. [file 41598_2022_7943_MOESM8_ESM.tif]
